# Intrinsic Self-Healing Chemistry for Next-Generation Flexible Energy Storage Devices

**DOI:** 10.1007/s40820-023-01075-9

**Published:** 2023-04-10

**Authors:** Xin Wan, Tiansheng Mu, Geping Yin

**Affiliations:** MIIT Key Laboratory of Critical Materials Technology for New Energy Conversion and Storage, School of Chemistry and Chemical Engineering, Harbin Institute of Technology, Harbin, 150001 People’s Republic of China

**Keywords:** Flexible energy storage, Intrinsic self-healing chemistry, Lithium-ion battery, Supercapacitor, Advanced characterizations

## Abstract

The booming wearable/portable electronic devices industry has stimulated the progress of supporting flexible energy storage devices. Excellent performance of flexible devices not only requires the component units of each device to maintain the original performance under external forces, but also demands the overall device to be flexible in response to external fields. However, flexible energy storage devices inevitably occur mechanical damages (extrusion, impact, vibration)/electrical damages (overcharge, over-discharge, external short circuit) during long-term complex deformation conditions, causing serious performance degradation and safety risks. Inspired by the healing phenomenon of nature, endowing energy storage devices with self-healing capability has become a promising strategy to effectively improve the durability and functionality of devices. Herein, this review systematically summarizes the latest progress in intrinsic self-healing chemistry for energy storage devices. Firstly, the main intrinsic self-healing mechanism is introduced. Then, the research situation of electrodes, electrolytes, artificial interface layers and integrated devices based on intrinsic self-healing and advanced characterization technology is reviewed. Finally, the current challenges and perspective are provided. We believe this critical review will contribute to the development of intrinsic self-healing chemistry in the flexible energy storage field.

## Highlights

- The introduction of self-healing mechanism into flexible energy storage devices is expected to solve the problems of mechanical and electrochemical performance degradation caused by mechanical deformation.
- Applications of different healing mechanisms and advanced characterization techniques in energy storage devices are summarized.
- The key challenges of self-healing in the field of flexible energy storage are pointed out, and the future research direction is prospected.

## Graphical abstract

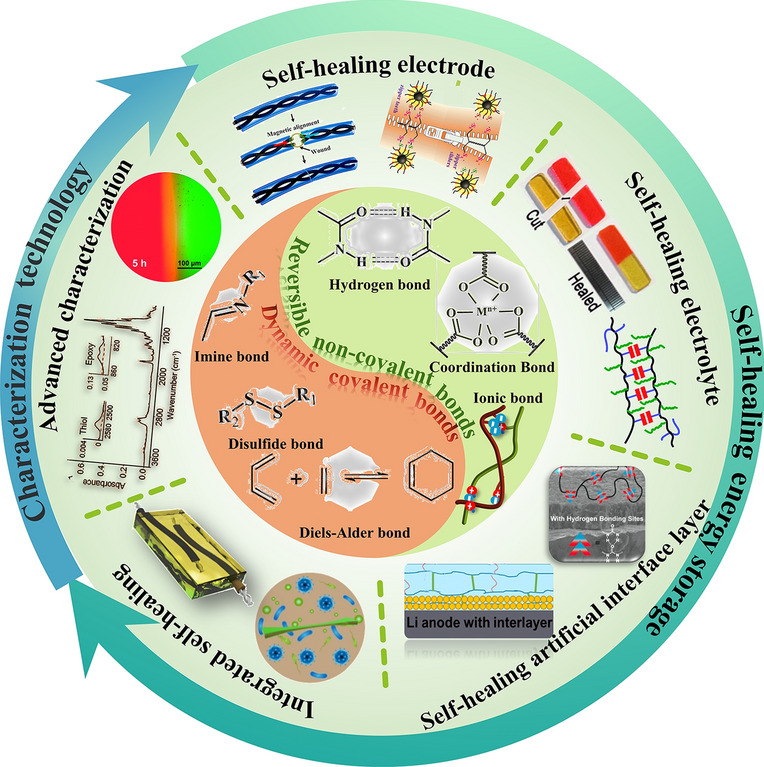

## Introduction

With the rapid progress of electronic technology, more and more portable electronic devices are developing toward the flexible wearable direction [1–6]. At present, achieving ultra-long standby time and the service life is one of the important research fields of flexible devices, which puts forward higher requirements for energy storage components [7–9]. Lithium-ion batteries (LIBs) and supercapacitors are the most commercially successful two classes of energy storage and conversion devices. LIBs possess high energy density and excellent stability performance [10, 11], and supercapacitors have ultra-high power density and long service life [12–14]. Therefore, they can complement each other with advantages, which have been widely applied in flexible devices. Currently, numerous design strategies for flexible energy storage devices have being explored such as one-dimensional (coaxial, spring and spine type), two-dimensional (sandwich, wave and *z* type) and three-dimensional (honeycomb, origami and paper-cut type) [7]. In practical applications, excessive repeated mechanical deformation will still lead to performance degradation or even failure of flexible LIBs and supercapacitors [15–21]. From the microscopic perspective, the phase transformation and volume fluctuation of the electrode materials can lead to the passivation, cracking and falling off of the electrode [7, 22, 23], which will deteriorate the device’s electrochemical performance, and such micro-damages are difficult to detect. From a macro-perspective, the special application environment makes the flexible energy storage device inevitably suffer some mechanical shock, perforation and wear during the long-term cycle, which eventually leads to performance failure and limited service life of energy storage devices [24–28]. Optimization of the electrochemical performance of flexible batteries or capacitors remains a key challenge.

In nature, many organisms have the ability to repair damage spontaneously, which is an important property for extending the lifespan of an organism. Similarly, some functional materials also have the function of self-healing, which are called self-healing materials, and they can repair the matrix damage caused by mechanical work [29–33]. Therefore, introducing smart self-healing materials into flexible batteries or capacitors is considered to be a very promising means to resist mechanical damage and prolong their life [21, 29, 34–36]. In 2001, White et al. [37] first proposed self-healing microcapsule based on embedded system. When the crack occurs, the siphon effect will promote the healing agent to flow from the capsule to the crack, contact with the catalyst and initiate monomer ring-opening polymerization, so as to solidify at the crack and prevent the crack from expanding. Microcapsule healing and subsequent 3D microvascular network healing depending on additional healing agents in the polymer are collectively referred to as extrinsic self-healing [38–43]. Extrinsic self-healing has the advantages of quick trigger response, high healing efficiency and simplicity. However, in the planning and manufacturing of extrinsic self-healing materials, it is necessary to consider whether microcapsules and micro-vessels carrying restorative agents have an impact on the function of the substrate. Therefore, extrinsic self-healing materials are mostly used in the fields of coatings, foam materials, ceramics and metal manufacturing, and progress in the field of energy storage is relatively slow. However, it cannot be ignored that the extrinsic self-healing system has shown unique advantages in the large-area damage caused by medium-energy impact such as impact and fatigue. Considering the high demand for flexible energy storage device packaging, the development of buried extrinsic self-healing sealant can better fill the research gap of packaging. To overcome these limitations of extrinsic self-healing, intrinsic self-healing was proposed, consisting of dynamic covalent bonds and reversible non-covalent bonds. The covalent or non-covalent bonds are introduced into the polymer by cross-linking, reversible chemical reactions and polymer diffusion, which endows the materials with the ability to be dynamically broken and re-assembled [44–46]. According to thermodynamic theory, the spontaneous behavior of chemical processes can be explained by the change of Gibbs free energy (Δ*G*) in the reaction. This means that the total energy of the process, (Δ*G* = Δ*H* − *T*Δ*S*) is less than 0, that is, the enthalpy decreases and the entropy increases in the reaction process, and the reaction is spontaneous [30, 47]. This kind of self-healing material has the advantages of mild repair conditions and repeatable repair [48, 49]. To adapt to the needs of the flexible electronic equipment, the combination of intrinsic self-healing and energy storage has gradually become a strategy to break through the bottleneck of electrochemical performance. To date, most advances about self-healing energy storage focus on the repair efficiency and electrochemical performance, while the properties of self-healing chemistry, repair mechanisms and advanced characterization techniques are also critical for the development of excellent self-healing materials.

In view of the critical importance of self-healing capability, we summarized the latest research progress of self-healing in the field of flexible energy storage. The part 2 of the review summarizes the self-healing mechanism and healing characteristics. The part 3 introduces the latest research progress of self-repair energy storage devices and evaluates the characteristics and limitations of different repair methods. The part 4 summarizes the advanced characterization technology involved in the development and application of self-healing materials. At the end of the last section, the challenges of self-healing are summarized and its application prospects are prospected.

## Intrinsic Self-healing Mechanism

Dynamic covalent and reversible non-covalent bonds are two research hot spots of intrinsic self-healing, belonging to dynamic chemistry essentially [50, 51]. Dynamic covalent bonds can be remodeled, repaired and regenerated through topological evolution at the molecular level under external energy and stimulus conditions [45, 52–57]. Therefore, dynamic polymer systems constructed by reversible covalent bonds are endowed with intelligent response properties. Non-covalent bonds are based on molecular interactions (such as hydrogen bond, ionic bond and coordination bond), which are dynamic reversible in nature and do not require external intervention, and can provide autonomous error correction capability of molecular recognition and recognition direction. Polymers containing reversible non-covalent bonds can be cross-restructured through the dynamic reversible characteristics of non-covalent bonds, and the matching degree between building elements can be flexibly controlled to control the performance of materials [58], so as to ensure that the designed molecular structure is the most advantageous species in thermodynamics, and obtain strong and self-healing molecular structure. Dynamic covalent bonds and reversible non-covalent bonds are parallel and complementary to each other in a sense, forming constitutional dynamic chemistry together. Therefore, coordinating the two types of properties to suit different application requirements is expected to further promote the self-healing field.

### Dynamic Covalent Bonds

Dynamic covalent bonds depend on the reversible formation and fracture of a fairly strong covalent bond within a molecule, and its fracture and reconstruction process is driven by thermodynamics [47]. Therefore, the thermodynamic equilibrium process of dynamic covalent networks is often much slower than the non-covalent bond self-assembly process, and the former usually requires the assistance of external conditions (such as heat, pH, light and catalyst). But importantly, it combines the error correction capability of reversible non-covalent bonds and the robustness of covalent bonds [51], which overcomes the shortcomings of permanent covalent and adds new functions. Chemical bonds such as Diels–Alder bond [59–63], disulfide bond [64–67] and imine bond [68–71] belong to the category of dynamic covalent bonds.

#### Diels–Alder Bond

**Fig. 1:**
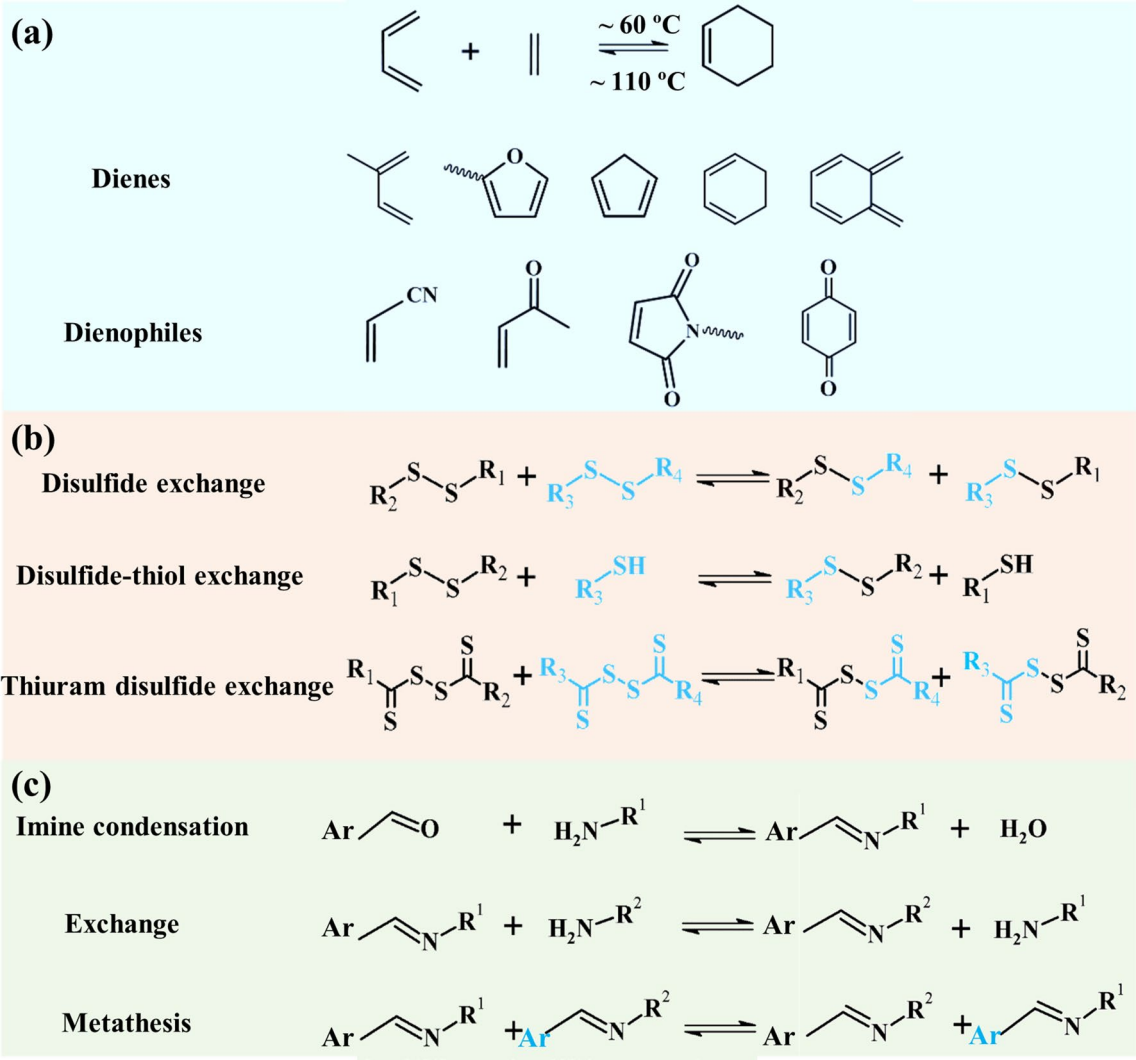
**a** Diagram of Diels–Alder reaction and common monomers. **b** Types of reversible redox exchange for disulfide bonds (reproduced with permission from Ref. [47]. Copyright 2018, Elsevier). **c** Three types of imine reactions (reproduced with permission from Ref. [84]. Copyright 2012, Royal Society of Chemistry)

The Diels–Alder (DA) reaction originated in 1928, but it was not until around 2000 that the self-healing chemistry depended on DA developed vigorously. DA reaction is a kind of dynamic covalent chemical reaction affected by temperature (Fig. 1a). The reactant in which DA reactions occur consists of two parts, one of which provides a conjugated diene, called dienes, and the two carbon–carbon double bonds of the diene must be in cis conformation. The other part provides unsaturated bonds, called dienophiles. At low temperature, [4 + 2] cycloaddition reaction occurs between dienes and dienophiles to form DA admixtures [72]. Under high temperatures, the DA addition can undergo reverse DA reaction (r-DA reaction), and the molecular chain breaks into dienes and dienophiles [73, 74]. This cyclic addition-reversible decomposition reaction is repeatable, so multiple cracks can be repaired in the same location by heating and cooling. DA reaction presents the advantages of relatively mild reaction conditions, fewer side reactions, no need for metal catalyst, fast kinetics and high yield [62, 75, 76]. Therefore, the repair process can be realized only by changing the temperature, which provides a feasible way to develop recyclable materials.

#### Disulfide Bond

A disulfide bond (S–S) is a type of covalent bond and occurs between two sulfur atoms. The average dissociation energy of S–S is approximately 240 kJ mol^−1^, which is lower than carbon–carbon (C–C) single covalent bond (346 kJ mol^−1^) [77, 78]. Therefore, S–S are very weak short bonds and require less energy to form. The S–S can form sulfhydryl group after breaking through reduction reaction. If the sulfhydryl group is oxidized, the S–S will be formed again. There are three main types of reversible redox exchange for S–S [47]: (1) disulfide exchange [79], (2) disulfide-thiol exchange [80] and (3) thiuram disulfide exchange [53]. The following set of chemical equation diagrams can graphically represent these mechanisms (Fig. 1b). The S–S can be recombined with the same or different sulfur atoms and can be broken and repaired many times at a lower temperature (compared with DA reaction), catalysts [81] or UV irradiation [82].

#### Imine Bond

The above-mentioned reversible covalent structures (DA and S–S) can prepare self-healing polymers with certain mechanical properties, but they often encounter problems such as complex preparation process, high temperature stimulation and poor recovery. Schiff base reaction, discovered by Hugo Schiff in 1864 [83], refers to the formation of imine bonds between amine derivatives and aldehydes or ketones. The class of condensation product has the general formula R^1^R^2^C=NR^3^, where R^1^ and R^2^ can be either (a) an aryl or alkyl group, (b) a mixture of the two or (c) a hydrogen atom with R^3^ an aryl or alkyl group (Fig. 1c) [84]. Imine bond (C=N) is a typical dynamic covalent bond, which is conducted under thermodynamic control. The presence of external factors can affect the equilibrium between imines and their corresponding precursors during the appropriate time, and the intermediate products with dynamic competitiveness will be replaced by the most stable products in thermodynamics. The synthesis conditions of imine bonds are diverse, and the target products with different properties can be obtained, such as self-healing and reprocessing polymers [85], degradable polymers and pH-responsive polymers [86, 87].

### Reversible Non-covalent Bonds

Non-covalent bonds have lower bond energy than dynamic covalent bond systems; therefore, it generally has higher repair efficiency. The interactions between non-covalent bonds tend to dissociate rather than break, showing infinite self-healing function. Different from the repair conditions of dynamic covalent bonds, non-covalent bonds achieve dynamic repairing equilibrium through molecular chain migration, identification and recombination. In essence, the result of this process is the spontaneous recovery of the mechanical properties of the repair area through the chain entanglement. Therefore, chain diffusion at the polymer–polymer interface is considered to be the main driving force for repair [30]. From the microscopic perspective of repair, the bonds in the polymer network must be dynamic under certain conditions. From the macroscopic perspective, the fracture surface of the polymer must be close enough to realize the repair process. Therefore, although the existence of non-covalent bonds is widespread, ordinary polymers containing non-covalent bonds may not be able to realize the repair process due to limited chain mobility and lack of bond recombination ability [88]. Reversible non-covalent bonds include all the weak interactions that occur between different atoms, such as hydrogen bond [89–95], ionic bond [96–98] and coordination bond [99].

#### Hydrogen Bond

**Fig. 2:**
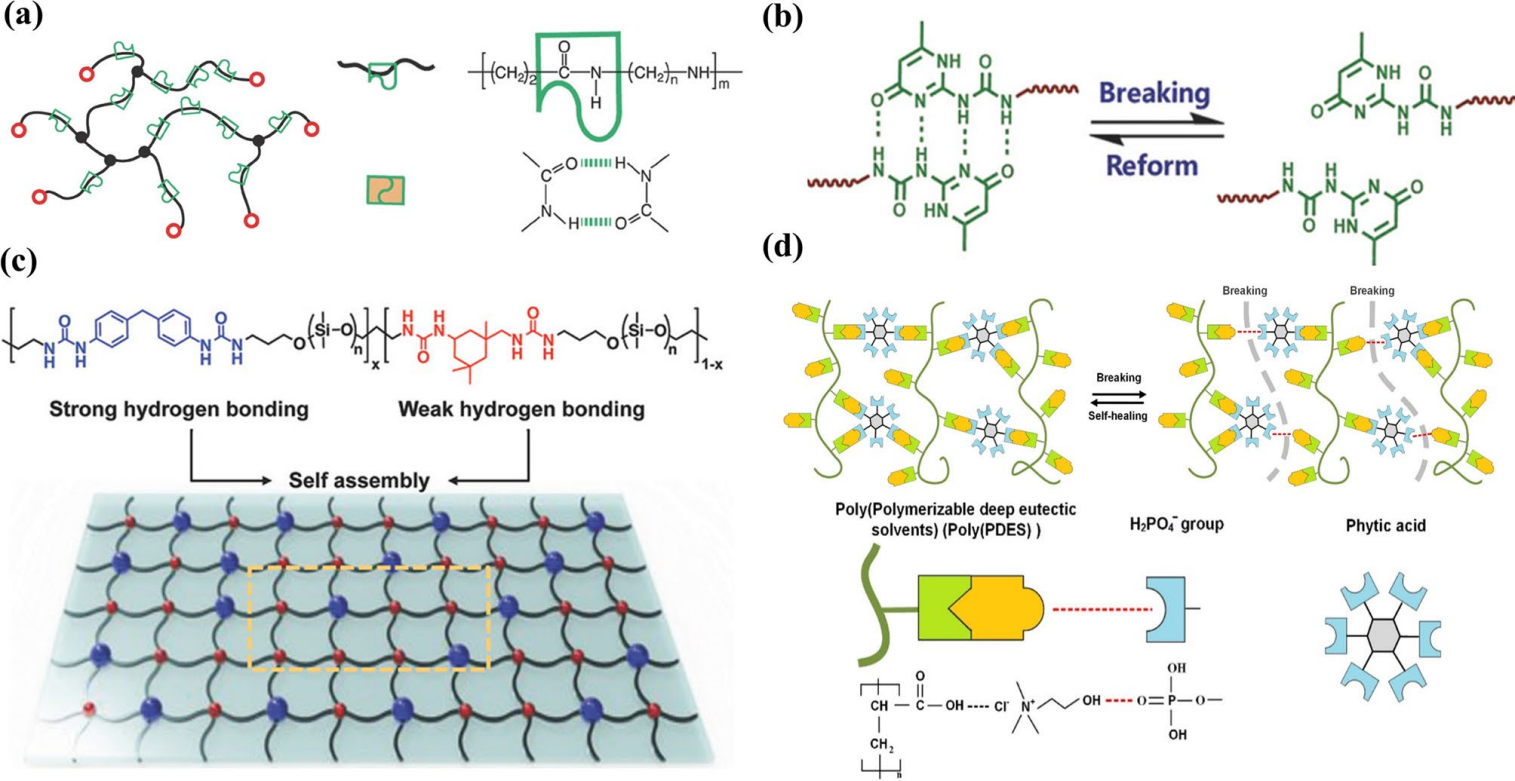
**a** Excellent self-healing elastomer with double hydrogen bond (reproduced with permission from Ref. [102]. Copyright 2017, WILEY–VCH). **b** Quadruple hydrogen of the ureido-pyrimidinone (reproduced with permission from Ref. [103]. Copyright 2018, WILEY–VCH). **c** Coexistence strategy of weak and strong hydrogen bonding (reproduced with permission from Ref. [105]. Copyright 2018, WILEY–VCH). **d** Self-repairing elastomers with multiple hydrogen bonds (reproduced with permission from Ref. [106]. Copyright 2020, Elsevier)

Hydrogen bond is a widespread supramolecular weak interaction with strong directivity and is very important position in chemistry, biology, physics and other fields since it is proposed and identified by Pauline in the 1930s. The mechanism is that hydrogen atoms and highly electronegative X atoms (such as O, N and F) are bonded together by covalent bonds, where X–H is called a proton donor. When X–H are near another Y atom (proton acceptor) with high electronegativity (O, N, F), the molecular structure of X–H…Y will spontaneously form, resulting in hydrogen bond. The bond energy of individual hydrogen bonds are relatively weak, ranging from 5 to 30 kJ mol^−1^ [100, 101]. The softness of a single hydrogen bond is related to the X–H dipole moment, the lone pair of electrons on the Y atom and the solvent state. It is worth noting that the superposition and synergy of multiple hydrogen bonds can obtain strong binding energy, for example, excellent self-healing elastomer for molecular integration of double hydrogen bond network (Fig. 2a) [102]. The ultra-high binding constant (6 × 10^7^ m^−1^) and superior bond energy (44 kJ mol^−1^) of the quadruple hydrogen bond in chloroform make it one of the most promising supramolecular building blocks for constructing fast self-healing materials (Fig. 2b) [103]. For multiple hydrogen bonds, the bond strength ranges from high dynamic bonds to quasi-covalent bonds [104]. The influence factors are closely related to the atom types, re-number of hydrogen bond, and the additional effects between adjacent donors and receptors. Polymers with weak hydrogen bonds have high viscoelasticity and weak mechanical toughness, and they cannot resist large deformation. Conversely, the strong hydrogen bonds can provide the high mechanical stability the weakened repair efficiency. Bao et al. [105] proposed a preparation strategy of self-healing materials cross-linked by hydrogen bonds with different strength. A series of polymers were synthesized through one-pot polycondensation by adjusting the proportion of the mixture. Such polymers with different strength can spontaneously form hydrogen bond cross-linking points. Strong hydrogen bond cross-linking can endow polymer with toughness and elasticity, while weak cross-linking can dissipate strain energy through efficient reversible bond fracture and reconstruction. Therefore, precisely controlling chemical molecular structure of polymer is an effective strategy to prepare strong and fast self-healing polymers (Fig. 2c-d) [105, 106].

#### Ionic Bond

**Fig. 3:**
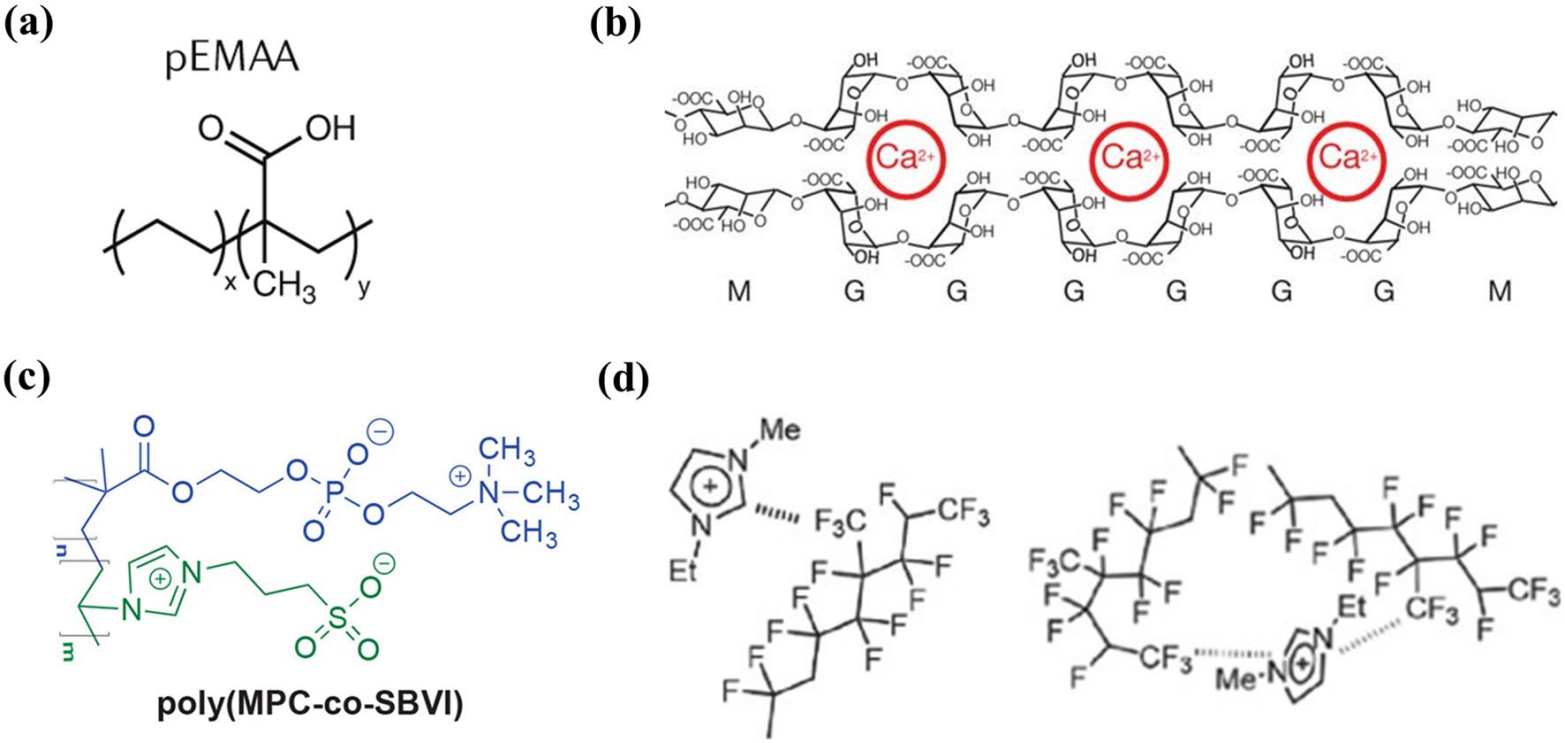
**a** Self-healing ionomer (pEMAA) (reproduced with permission from Ref. [108]. Copyright 2020, Springer Nature Limited). **b** Calcium ion (Ca^2+^) cross-linked elastic hydrogels (reproduced with permission from Ref. [109]. Copyright 2012, Nature Publishing Group). **c** Solvated ion gel electrolytes (reproduced with permission from Ref. [110]. Copyright 2019, American Chemical Society). **d** Ion–dipole interactions (reproduced with permission from Ref. [111]. Copyright 2016, WILEY–VCH)

Compared with hydrogen bonds, ionic bonds have greater binding energy and higher stability. There is electrostatic interaction between atoms or groups with opposite charges. When they are close to each other, electrostatic attraction is generated to form ionic bond. Unlike hydrogen bond, ionic bond has not directionality and non-saturation. As long as space conditions are available, an ion can be combined with many ions with opposite charge, that is, positive and negative ions have the same ability to attract ions with opposite charge in any space direction [107]. Ionic polymers are the first products that are proved to have restorative properties. For example, poly(ethylene-co-methacrylic acid) (pEMAA) maintains the intermolecular ionic attraction under environmental conditions and projectile puncture test (Fig. 3a) [108]. In addition, there are calcium ion (Ca^2+^) cross-linked elastic hydrogels (Fig. 3b) [109], solvated ion gel electrolytes (Fig. 3c) [110] and ionic conductor based on ion–dipole interaction (Fig. 3d) [111].

#### Coordination Bond

**Fig. 4:**
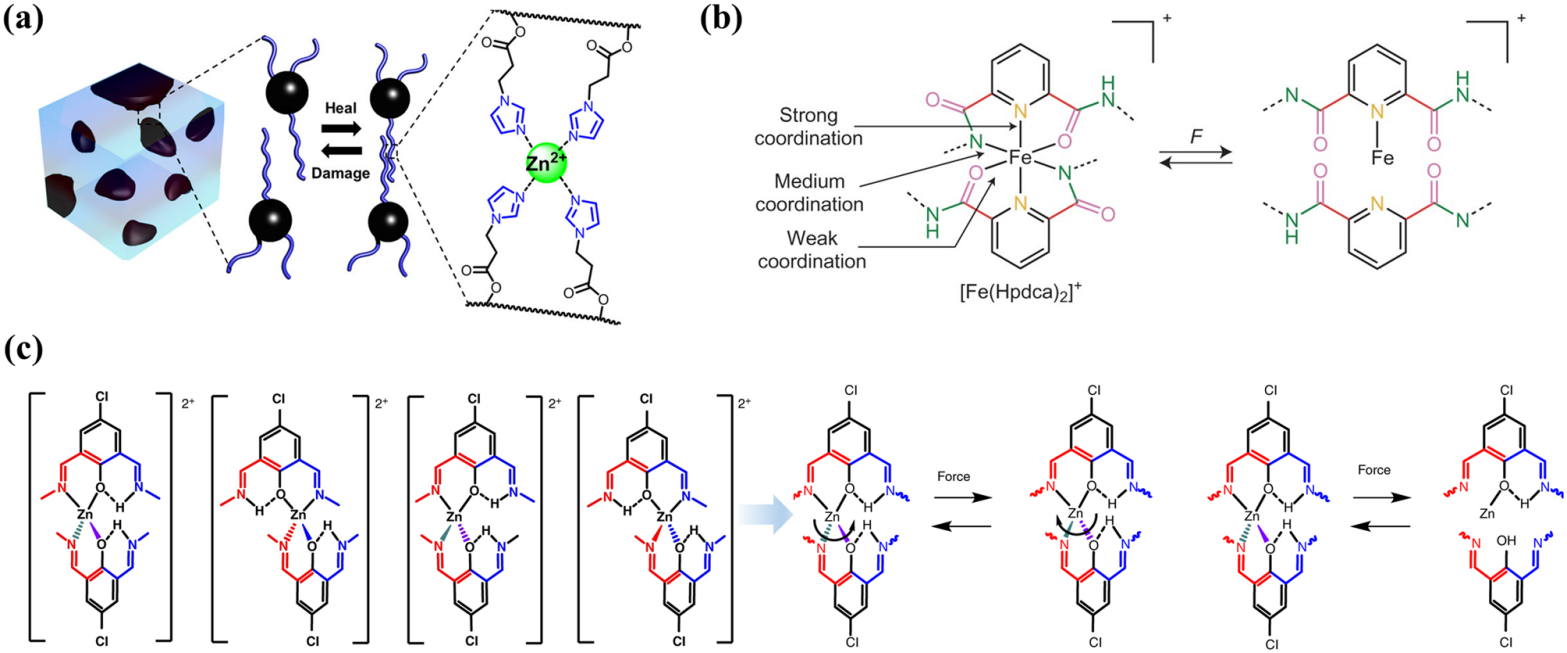
**a** Dynamic zinc–imidazole interactions (reproduced with permission from Ref. [116]. Copyright 2014, American Chemical Society). **b** Different strengths of metal coordination bonds (Fe(III)–Npyridyl, Fe(III)–Namido and Fe(III)–Oamido) (reproduced with permission from Ref. [117]. Copyright 2016, Nature Publishing Group). **c** Possible stereochemical structures complex and energy dissipation process for [Zn(Hbimcp)_2_]^2+^ (reproduced with permission from Ref. [118]. Copyright 2019, The Author(s), Springer Nature)

Metal–ligand (M–L) coordination bonds are very unique non-covalent interactions formed by the coordination between metal ions that has the ability to receive lone pair electrons and a ligand that can provide electrons [112]. The metal ions (Cu^2+^, Zn^2+^, Al^3+^ and Fe^3+^) are generally used as metal coordination bonds, and common ligands include pyridine, imidazole and carboxylic acid. Among many reversible non-covalent bonds, the coordination bonds can greatly affect the physicochemical properties of self-healing polymers, from weakly viscous fluids to strong elastic solids [113–115]. First of all, the formation of coordination bonds is a thermodynamic spontaneous process of enthalpy reduction and entropy increase, which is conducive to the material preparation and self-repair without any external stimulus. For example, Zn^2+^-imidazole, as a healing primitive, has relatively small binding constant and strong dynamic M–L complex exchange (Fig. 4a) [116]. Secondly, by adjusting the M–L type, the combined coordination bond strength can reach 25–95% of covalent bonds, which facilitates the acquisition of high toughness/modulus and fast self-healing materials. For example, a strong metal ligand binding site is placed near the weak binding site of the ligand 2,6-pyridinedicarboxamide, resulting in a highly dynamic M–L interaction of both strong and weak. The fracture and re-formation of weaker bonds achieve energy dissipation and self-healing after damage, while the stronger interaction keeps metal ions near the ligand and promotes the rapid re-formation of bonds (Fig. 4b) [117]. Moreover, in spite of their highly dynamic nature, they are thermodynamically stable. Thermodynamic stability focuses on whether the generated substances can be converted into other products, i.e., Δ*G* of the reactions. Kinetic stability is concerned with the reaction rate. The mechanical properties of the polymer are determined by the thermodynamic stability, and the self-healing rate is determined by the dynamic instability of the cross-linking site. To better adapt to the application requirements, the current materials need to balance the relationship between mechanical properties and dynamic healing. The design of the cross-linking site with thermodynamic stability and dynamic instability is critical to the realization of materials with high toughness/modulus and self-healing properties [112]. Thus, by fine-tuning the thermodynamic and kinetic properties of coordination bonds, it is possible to obtain mechanically robust self-healing polymers with diverse compositions (Fig. 4c) [118].

## Intrinsic Self-healing Chemistry in Energy Storage Devices

Energy storage devices are increasingly being incorporated into flexible design concepts to meet the requirements of compact, lightweight and comfortable electronics, and some special bending conditions [119–121]. However, under long-term bending and buckling stress, the flexible energy storage devices will inevitably appear in mechanical fatigue and damage. Therefore, flexible devices with self-healing characteristics are considered to be the next-generation energy storage development trend. This chapter mainly introduces the research progress of intrinsic self-healing flexible energy storage devices, including self-healing electrode, self-healing electrolyte, self-healing artificial interface layer, and integrated self-healing.

### Self-healing Electrode

The main damage at the electrode is micro-crack caused by mechanical stress, volume fluctuation or lattice mismatch [122]. For example, silicon and sulfur electrodes have attracted much attention owing to due to their ultra-high theoretical capacities [123–125]. However, electrode fracture during repeated bending, volume expansion/contraction, and severe shuttle effect of sulfide have triggered multiple decay mechanisms [126]. Incorporating the self-healing polymer as substrates or binders into the electrode provides a simple versatile way to manufacturing self-healing electrodes.

#### Self-healing Substrate

**Fig. 5:**
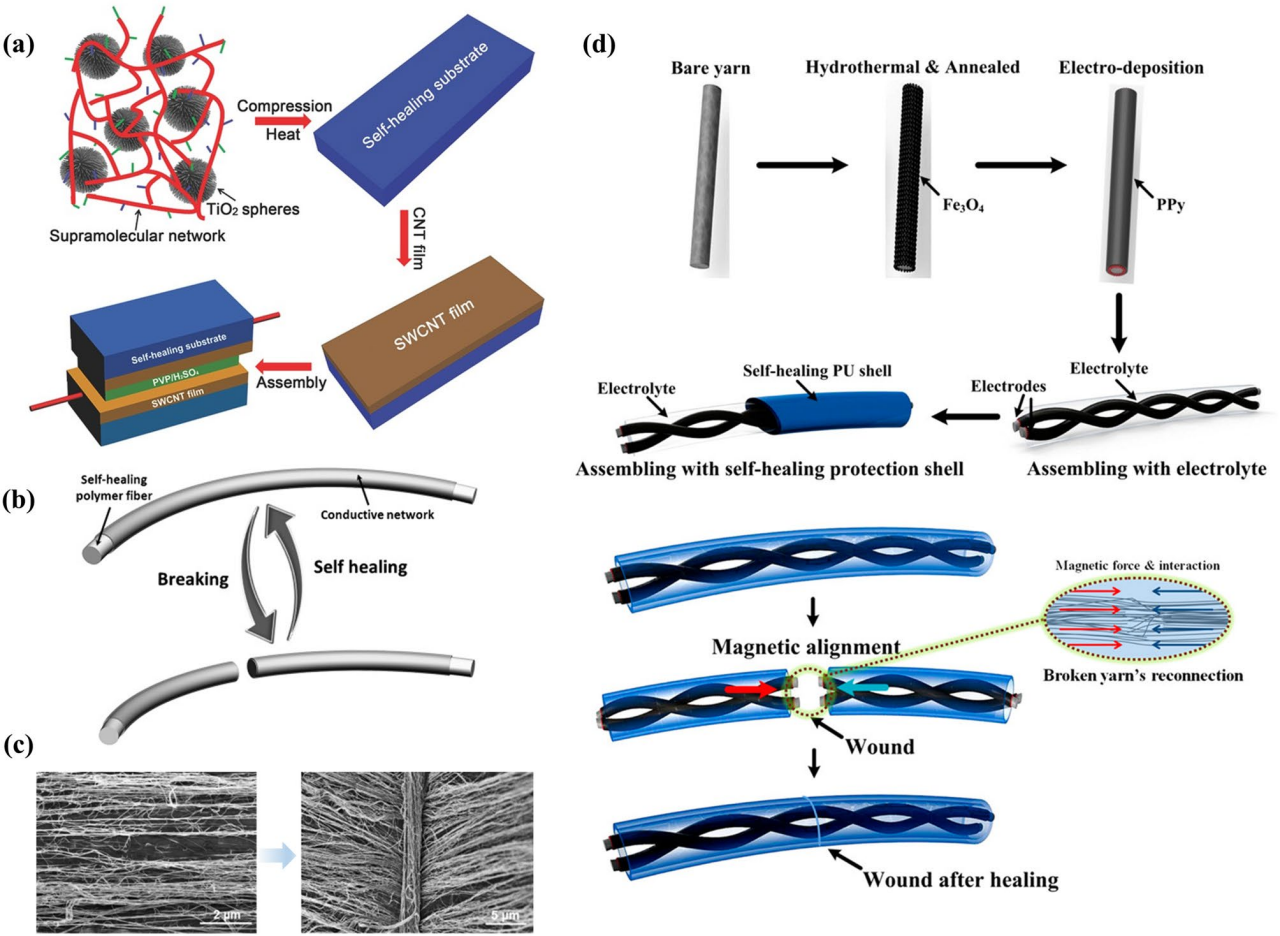
**a** Description of self-healing substrate (reproduced with permission from Ref. [127]. Copyright 2014, WILEY–VCH). **b** Schematic illustration of the self-healing wire. **c** Scanning cross sections of self-healing (reproduced with permission from Ref. [128]. Copyright 2014, WILEY–VCH). **d** Design and manufacturing process flow of the magnetic-assisted self-healable supercapacitor (reproduced with permission from Ref. [129]. Copyright 2015, American Chemical Society)

Introducing repairable substrates into electrodes is a common means to realize self-healing electrodes. Wang et al. [127] fabricated electrodes by depositing single-walled carbon nanotubes (SWCNT) onto self-healing substrates. The substrate is composed of uniformly dispersed TiO_2_ particles and dynamically reversible hydrogen bond polymer with low glass transition temperature (*T*_g_). Upon being subjected to mechanical damage, lateral movement of the self-healing composite layer brings the separated areas of the SWCNT layer into contact, thus restoring the configuration and conductivity of the device (Fig. 5a). The single repair force is weak and incapable of superior self-healing properties, and the synergistic multiple effects are more conducive to achieving high healing effectiveness. Sun et al. [128] used a similar method to scroll the aligned CNT layer closely and uniformly onto SHP fibers (Fig. 5b). When the two fractured parts re-contact, the hydrogen bond will re-form between the self-healing fibers, and the collective van der Waals force effect of aligned carbon nanotubes with large density will produce a tough adhesion. The cross-sectional scanning electron microscope (SEM) confirmed that the fibers had undergone structural self-healing after fracture (Fig. 5c). The effective combination of hydrogen bond and van der Waals force can restore the mechanical and electrical properties of the damaged electrode and improve the ductility of the electrode, simultaneously. The above-mentioned healing forms are limited to simple mechanical recovery, and the reconstruction of conductivity after damage is the critical factor to determine the charging/discharging performance of supercapacitors. Therefore, supercapacitors with mechanical and electrical self-healing characteristics are an excellent solution to this challenge. Zhi et al. [129] proposed a magnetic (Fe_3_O_4_)-assisted self-healing strategy depended on substrate repair (Fig. 5d). A carboxylated self-healing polyurethane (PU) is used as the shell and is encased with a polypyrrole electrode containing magnetic Fe_3_O_4_. The strong magnetic force between the broken interfaces facilitates the re-connection of yarn electrodes, thus providing an effective strategy for restoring electrical conductivity.

The electrode damage–repair technology based on self-healing substrate can effectively restore the electrode performance by re-contacting the active material. However, there are still challenges as follows: (1) The insufficient interaction between active materials and substrates, the instability of the interface between the electrode and the substrate will limit the repair efficiency. (2) Although the self-healing substrate can effectively drive the electrode repair, the simple physical contact at the damaged interface results in the limited healing ability. With repeated damage, the healing reliability will gradually decrease.

#### Self-healing Binder

Another strategy to repairing electrode is self-healing binder. The binder role in the electrode is to make electrode components effectively adhere to current collector during battery operation [130]. Therefore, the self-healing properties of the binder are expected to take key part in the long-term cycling stability of flexible energy storage devices [131–134]. Compared with the healing substrate, the self-healing binder eliminates the complicated electrode preparation step, such as the entanglement and deposition of active materials on the substrate.

**Fig. 6:**
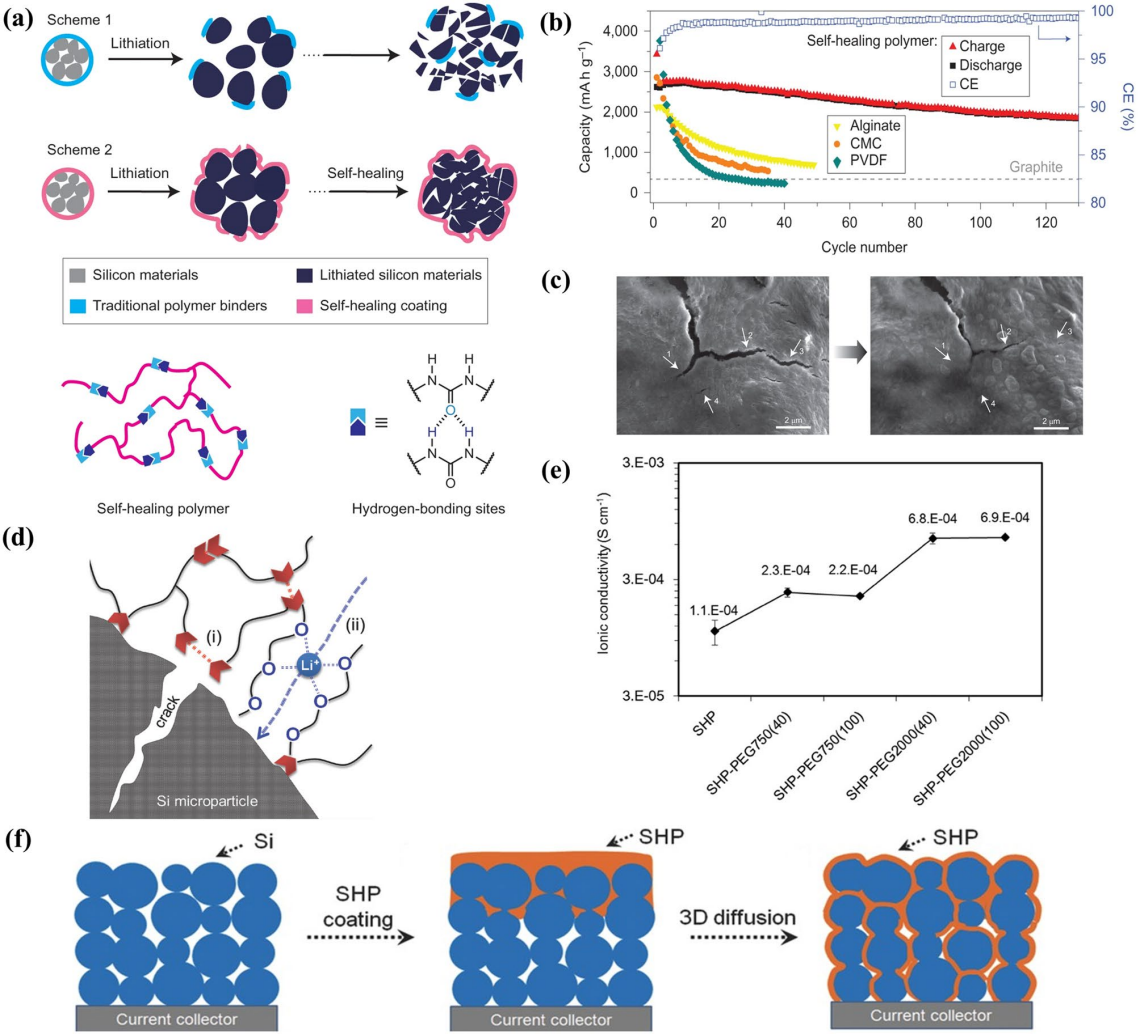
**a** Schematic diagram of conventional binders and self-healing binders on Si. **b** Comparison diagram of electrochemical performance. **c** SEM images of self-healing (reproduced with permission from Ref. [135]. Copyright 2013, Nature Publishing Group). **d** Schematic diagram of PEG group promoting Li^+^ conduction. **e** Ionic conductivity of self-healing binders with different molecular weights and mass fractions of PEG (reproduced with permission from Ref. [136]. Copyright 2018, WILEY–VCH). **f** Schematic design of Si-SHP electrodes (reproduced with permission from Ref. [137]. Copyright 2015, WILEY–VCH)

Researchers have achieved many outstanding results in self-healing binders over the past few years. Wang et al. [135] first reported the self-healing silicon (Si) electrodes by coating with a thin layer of double hydrogen bond-directed polymer (Fig. 6a). Amorphous polymer with low *T*_g_ and abundant hydrogen bonds was designed by controlling the reaction conditions, so that the polymer chains at the fracture interface can be quickly re-arranged, approached and mixed. Therefore, the electrode showed excellent cyclic electrochemical stability (Fig. 6b), contrast to Si anodes containing polyvinylidene difluoride (PVDF), carboxymethyl cellulose (CMC) and alginate. SEM images exhibit that the micro-cracks caused by lithiation/de-lithiation can be repaired at room temperature for 5 h (Fig. 6c). The influence of Li^+^ transport on battery energy density cannot be ignored while realizing self-healing. Munaoka et al. [136] introduced polyether units (PEG) into self-healing polymer (SHP) (Fig. 6d). The lone electrons on the polyether chain can be continuously complexed/recombined with Li^+^ to assist the Li^+^ transport in the matrix and improve the interface between Si and electrolyte. The electrochemical impedance results show that all PEG-modified films possess higher Li^+^ transport capability than SHP (Fig. 6e). It is worth noting that these two healing electrodes are coated with self-healing polymer on the electrode surface. It is difficult to penetrate or unevenly distributed for thicker electrode, which will cause the following disadvantages: (1) Larger cracks will form on unprotected Si. (2) SHP is difficult to diffuse into the generated cracks, resulting in poor repair efficiency. By repeatedly coating under heating condition (120–150 °C), the 3D distribution of SHP into Si particle layer reduces the diffusion lengths of SHP at the site of injury, thus providing an option for faster healing response (Fig. 6f) [137].

**Fig. 7:**
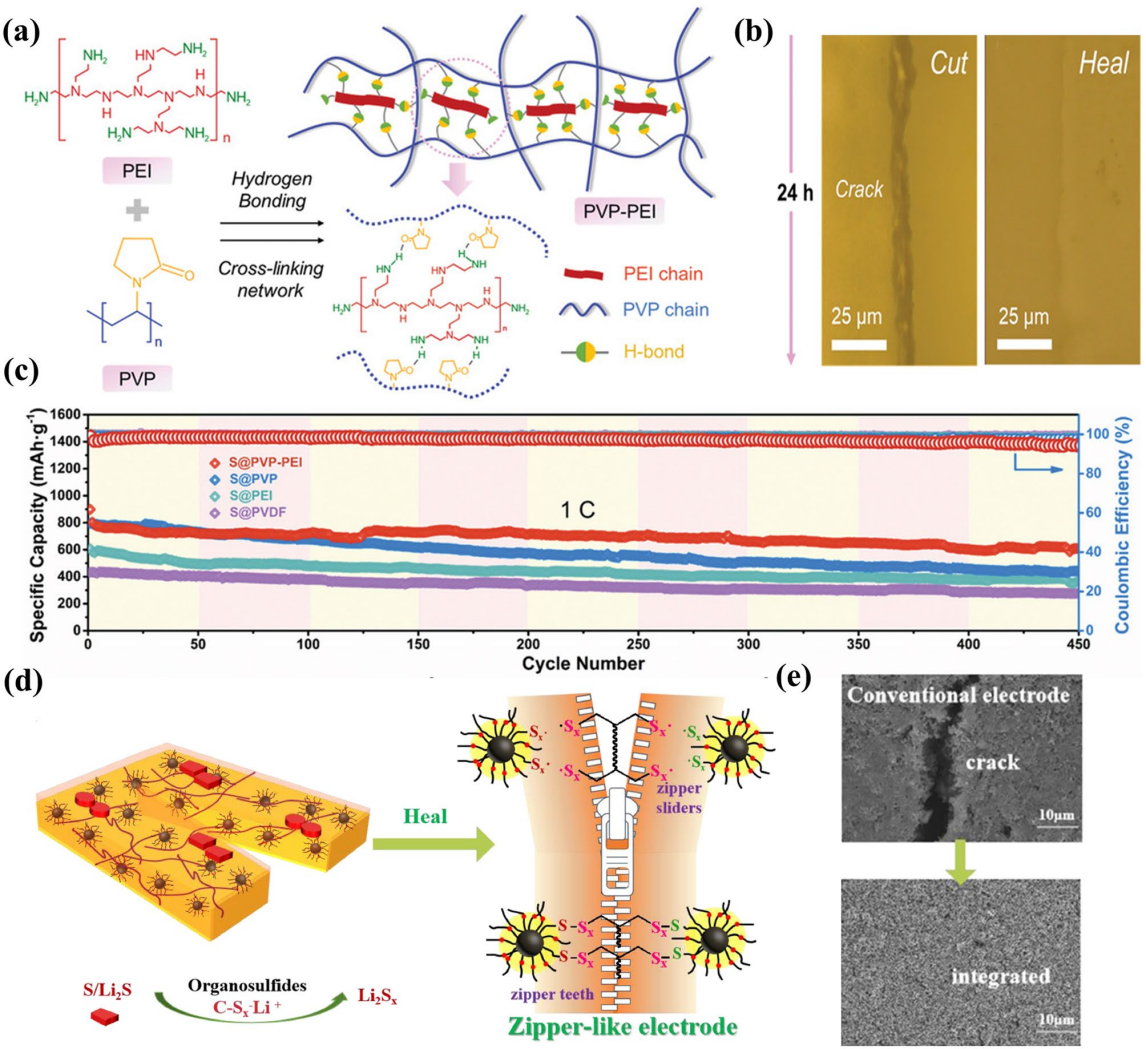
**a** Schematic of PVP–PEI polymer. **b** Experiment of damage/healing. **c** Electrochemical properties of S electrodes with different binders at 1C (reproduced with permission from Ref. [138]. Copyright 2021, WILEY–VCH GmbH). **d** Schematic of zipper-like electrode. **e** Comparison of morphology between conventional sulfur electrode and zipper-like sulfur electrode (reproduced with permission from Ref. [139]. Copyright 2020, Elsevier)

Self-healing polymers also have an important effect on improving the electrochemical performance of sulfur cathodes. Gao et al. [138] reported a self-healing polyvinylpyrrolidone–polyethyleneimine (PVP–PEI) binder cross-linked by hydrogen bonds (Fig. 7a). On the one hand, a large number of carbonyl groups (C=O) in PVP and amino groups (-NH_2_) in PEI can immobilize polysulfides and promote their redox conversion kinetics. On the other hand, -NH_2_ group can form a dynamic hydrogen bond network with C=O group and endow the binder with healing ability. The damage/healing experiment shows that the crack almost closed after 24 h (Fig. 7b). Importantly, the lithium–sulfur (Li–S) battery with PVP–PEI binder shows excellent cycle stability (the average capacity decay rate of each cycle after 450 cycles at 1 C is 0.0718%) (Fig. 7c). Generally, the binder amount only accounts for 1.5–3 wt% of the active substance in the electrode, which cannot effectively maintain the electrode structure and self-healing effect. Apart from the binder, the modification of other electrode components is another way to enhance the repair efficiency and battery cycle stability. Zeng et al. [139] designed a zipper-like sulfur electrode using sulfur nanocomposites graft organic polysulfide (-SX-) chains onto organic polysulfide (-SX-) polymer binder (PSPEG) and carbon host (CPS/S) as materials (Fig. 7d). The -SX- chains on/within the carbon matrix or binder can not only act as redox medium to control the phase transfer between S/Li_2_S and polysulfide, but also play the role of “zipper teeth” and “zipper sliders,” to spontaneously repair the damage of sulfur electrode. SEM images show more stable mechanical and electrical connections between zipper-like sulfur electrode than conventional sulfur electrode (Fig. 7e).

At present, the research on the electrode micro-cracks (mainly referring to the breakthrough of the elastic strain limit of the active material) has made some achievements, and the corresponding healing mechanism and related performance are summarized in Table 1. It is worth noting that how to efficiently integrate flexibility and electrochemical reversibility of self-healing materials requires further exploration. Some technically unsolved problems are briefly summarized as follows: (1) The matching relationship between the rigidity of electrode active materials and the flexibility of repair materials needs to be further analyzed. Balancing rigidity and flexibility are one of the key factors to improve the mechanical and electrochemical performance of self-healing electrodes by rational design of electrode structures and compositions. (2) The active self-healing materials in the electrode are still very limited, only a small amount of binder and substrate, which directly leads to insufficient repair capability and cannot meet the actual working conditions of batteries. Under the premise of considering the specific capacity and cycle life of batteries, it is an urgent challenge to develop an efficient self-healing electrode system. (3) Electron and ion transport kinetics of electrodes are a key factor affecting the electrochemical performance, which is paid much attention in previous research. Therefore, novel functional materials with rapid self-healing ability, good flexibility and excellent electrochemical activity remain to be developed.

**Table 1:** Comparison of healing efficiency and electrochemical performance of different electrodes

Electrode	Healing mechanism	Damage/healing method (time)	Electrochemical performance	References
Si@Fe-PDBP@pH10	Coordination bond	Scratch/healing (24 h)	74.6% (200th)	[131]
SiMP	Hydrogen bonding	Electrode morphology (5 h)	80% (90th)	[135]
Si-SHP-PEG	Hydrogen bonds	Scratch/healing (3 h)	80% (150th)	[136]
Si@SHPET	Hydrogen bond	Cut/healing (4 h)	58.4% (60th)	[95]
Si@PAA-UPy	Quadruple hydrogen bonding	Cut/healing(/)	62.89% (110th)	[103]
Si/PAA-BFPU	Disulfide bond	Scratch/healing (1 h)	97.0% (100th)	[140]
Si@CMC-CPAM	Electrostatic interactions	/	50.40% (100th)	[141]
S-PSPEG	Disulfide bond	Cut/healing (0.5 h)	812 mAh g^−1^ (300th)	[139]
S@PVP–PEI	Hydrogen bonds	Cut/healing (2 h)	880.1 mAh g^−1^ (150th)	[138]

### Self-healing Electrolyte

Electrolyte, as a main component of energy storage device, plays an important role in conducting ions and participating in redox reactions on the surface of anode/cathode electrodes. Compared with liquid electrolytes, polymer electrolytes have better mechanical strength and safety [142–145]. However, polymer electrolytes without excellent fluidity are difficult to heal themselves when cracks and fractures caused by external forces such as stretching, twisting and bending [146–149]. The development of a flexible self-healing electrolyte capable of spontaneously repairing damage is essential to improve the electrochemical performance and reliability of energy storage devices [150].

#### Self-healing Electrolyte Based on Non-covalent Bonds

**Fig. 8:**
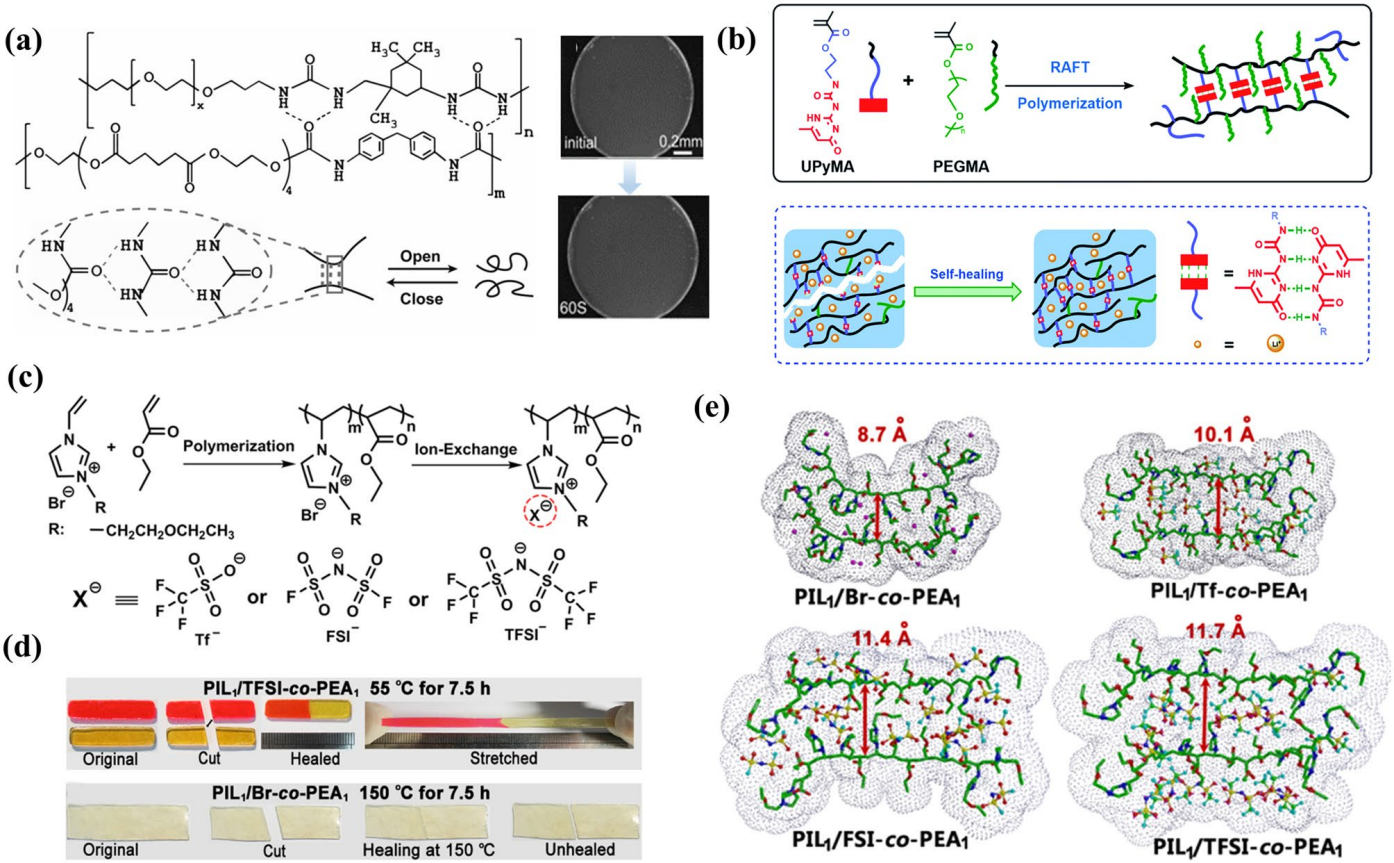
**a** Schematic diagram of solid polymer electrolytes with rigid–soft co-stability and self-healing experiment (reproduced with permission from Ref. [151]. Copyright 2019, WILEY–VCH). **b** Schematic diagram of synthesis and self-healing mechanism of brush-like copolymer (reproduced with permission from Ref. [153]. Copyright 2018, Royal Society of Chemistry). **c** Schematic illustration of the synthesis of the PIL-co-PEA copolymers paired with Br^−^, Tf^−^, FSI^−^ or TFSI^−^ counteranions. **d** Cutting/healing experiments of different counteranions. **e** Structure/conformation of different counteranions binding simulated by density functional (reproduced with permission from Ref. [154]. Copyright 2018, American Chemical Society)

Wu et al. [151] used amino-terminated poly(ethylene glycol) (NH_2_-PEG-NH_2_) as supramolecular skeleton and elastic thermoplastic polyurethane (TPU) cross-linking to prepare solid polymer electrolyte with self-healing and rigid–soft coexisting stability. Through the intramolecular hydrogen bonding between the urea group and the ester group, the electrolyte presents rapid self-healing (60 s) at the molecular level (Fig. 8a). Compared with single or double hydrogen bonds, multiple hydrogen bonds have a stronger recognition effect and can form a more powerful association. In 1996, the ureido-pyrimidinone (UPy, DADA four hydrogen bond) unit was first reported by the Meijer' group [152]. The ultra-high binding energy of quaternary hydrogen bonds and various binding methods have made the UPy system attract more and more attention. Zhou et al. [153] developed a brush-like polymer electrolytes (PEs) by using a UPy-containing monomer (2-(3-(6-methyl-4-oxo-1,4-dihydropyrimidin-2-yl)ureido)ethyl methacrylate) (UPyMA) and poly(ethylene glycol) methyl ether methacrylate (PEGMA) (Fig. 8b). Firstly, PEG side chains are expected to provide excellent ionic conductivity. Secondly, physical cross-linking can be achieved by connecting the UPy part through hydrogen bonds, thereby forming a flexible and fast self-healing PE. Their synergy can provide the electrolyte with fast intrinsic healing efficiency and high ductility. The above-mentioned polymer usually involves complex experimental design to achieve the dual effects of electrochemical stability and healing ability. Apparently, using reversible interactions controlled by their inherent chemical properties to fabrication self-healing materials is highly desirable because of the absence of additional groups that may adversely affect their function [154].

Ionic liquids (ILs) composed of ions and appears liquid at room temperature, having garnered increasing interest as potential electrolyte materials due to high ionic conductivity (1–10 mS cm^−1^), wide electrochemical window (3–5 V), high chemical and thermal stability, and non-flammability [110, 155–157]. Polyionic liquids (PILs) are formed by polymerizing ionic liquid monomers, which not only inherit the advantages of ILs, but also have the inherent polymers characteristics, and present superior processability and spatial controllability, thus overcoming the poor mechanical properties of ionic liquids. The inherent characteristics of ILs and PILs are the electrostatic interaction between the polymer backbone or ions with counterions (cation/anion pairs), acting as a physical cross-linking point and endowing material self-healing properties. Guo et al. [154] discovered that the counteranions can effectively mediate the healing properties of PIL copolymer (Fig. 8c). After the Br^−^ counteranions are exchanged with a larger size counteranions (TFSI^−^), the PIL copolymer gains healing capability by pairing with the Br^−^ counteranions. The PIL copolymer paired with TFSI^−^ showed excellent intrinsic healing properties at 55 °C, whereas no healing phenomenon was observed at 150 °C when Br^−^ was selected as the counteranions (Fig. 8d). According to density functional theory, because the larger counteranions exhibit less surface charges and longer distances with the imidazolium cations, the polymer chains in copolymers are loosely bonded, which has strong mobility and reversible electrostatic interaction (Fig. 8e). The strong electrostatic effect of Br^−^ can bind the polymer chain tightly, so that the rigid copolymer loses its repairing properties. The research shows that larger counterions can form loose ionic aggregates, resulting in weak physical cross-linking and decreased *T*_g_, maintaining a certain degree of dynamic self-healing, but poor mechanical strength [158]. The decreased ion size has the opposite property. Therefore, the *T*_g_ and ion aggregation degree of PILs can be systematically adjusted by optimizing components ratios to obtain desirable mechanical and healing performance.

#### Self-healing Electrolyte based on Dynamic Covalent Bonds

**Fig. 9:**
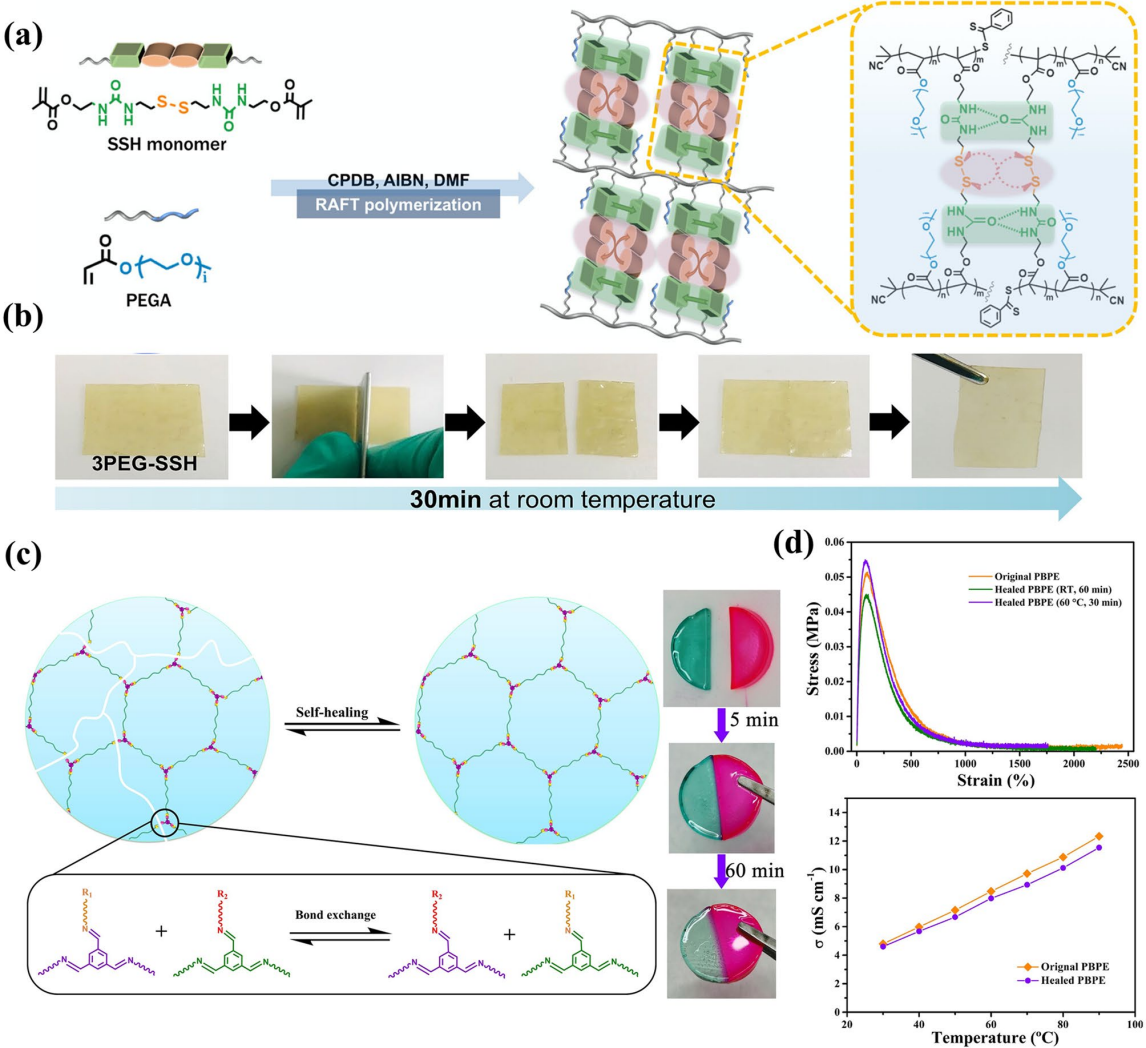
**a** Schematic illustration of the formation of PEG-SSH. **b** Self-healing experiment (reproduced with permission from Ref. [159]. Copyright 2020, American Chemical Society). **c** Self-healing PBPE containing imine bond and self-healing experiment. **d** Mechanical properties and ionic conductivity (reproduced with permission from Ref. [160]. Copyright 2021, Elsevier)

Besides, the self-healing electrolytes based on dynamic covalent bonds have also made some progress. Jo et al. [159] fabricated self-healing electrolyte (PEG-SSH) by employing reversible addition–fragmentation chain transfer (RAFT) polymerization of poly(ethylene glycol) methyl ether acrylate (PEGA, *M*_n_ = 480) and cross-linker consisting of hydrogen bond and disulfide bond (Fig. 9a). The re-formation of disulfide and hydrogen bonds at the broken interface restores the electrode to its original state. The damaged surfaces spontaneously healed without any external stimulation after 30 min (Fig. 9b). Deng et al. [160] prepared an innovative polymer electrolyte (PBPE) based on highly reversible imine bond (C=N). PBPE was synthesized from poly(ethylene glycol) diamine (NH_2_-PEG-NH_2_) and benzene-1,3,5-tricarbaldehyde (BTA) by Schiff base reaction (Fig. 9c). The C=N bond is highly reversible and capable of rapid bond exchange. The reversible fracture and recombination of C=N bond endows PBPE have good self-healing performance within 1 h. After self-healing, PBPE has the same ionic conductivity and mechanical properties as the original PBPE (Fig. 9d).

From the properties analysis of the self-healing electrolytes (Table 2), it can be concluded that the overall mechanical strength is positively correlated with the reversible bond energy, but the enhanced mechanical strength will reduce the repair efficiency, and the improved bond energy is often carried out by a complex experimental process. However, self-healing ILs and PILs with high ionic conductivity have the defects of low *T*_g_ and high viscosity at room temperature. In addition, unlike other dynamic covalent bonds, self-healing materials with higher bonding energy of disulfide and imine bonds can be obtained by adjusting the compositions and have higher healing efficiency at mild temperatures without external stimulation. Therefore, the ideal electrolytes materials with high ionic conductivity, strong mechanical properties and fast self-healing properties can be realized by screening the dominant component properties of different dynamic bonds and adding complementary components.

**Table 2:** Properties comparison of different self-healing electrolytes

Electrolytes	Healing mechanism	Damage/healing method	Healing time	References
CPSHPE	Quadruple hydrogen bonding	Cut/healing	3 h (60 °C)	[143]
PEO@BPIL	Ionic bond	Scratch/healing	15 min (60 °C)	[145]
PVA/Zn(CF_3_SO_3_)_2_	Hydrogen bond	Scratch/healing	30 min (room temperature)	[146]
PUSPEs	Disulfide bond	Cut/healing	12 h (60 °C)	[150]
SHSPEs	Hydrogen bonding	Cut/healing	60 s (room temperature)	[151]
PEGMA: UPyMA	Quadruple hydrogen bonding	Cut/healing	2 h (30 °C)	[153]
PIL	Ionic bond	Cut/healing	7.5 h (55 °C)	[154]
poly[BDI][Tf_2_N][CF_3_SO_3_]-50	Ionic bond	Scratch/healing	1.5 h (50 °C)	[158]
PEG-SSH	Disulfide bonds/hydrogen bonds	Cut/healing	2 h (30 °C)	[159]
PBPE	Imine bonds	Cut/healing	1 h (room temperature)	[160]

### Self-healing Artificial Interface Layer

During the first charge/discharge of liquid–based electrolyte battery, electrode material can react with electrolyte at the solid–liquid interface, forming a passivation layer covering the surface of electrode, which is called solid electrolyte interface (SEI) [161]. On the one hand, the SEI can prevent the further decomposition of electrolyte components; on the other hand, it can effectively prevent the co-embedding of solvent molecules to avoid the damage of electrode materials. However, naturally grown SEIs are fragile and uneven. Due to the high activity and uneven deposition/dissolution of lithium (Li), the growing Li dendrites can puncture the already formed SEI, leading to continued SEI formation and electrolyte consumption [162–164]. Studies show developing of high-quality self-healing artificial SEI can buffer surface changes and inhibit dendrite growth. Herein, we mainly discuss the self-healing artificial SEI of Li metal anode. Due to its extremely high theoretical specific capacity (3,860 mAh g^−1^) and lowest electrochemical potential, Li metal is considered to be one of the most promising anodes for next-generation high specific energy battery. However, commercial application of Li still faces many challenges caused by high activity and uneven deposition/dissolution.

**Fig. 10:**
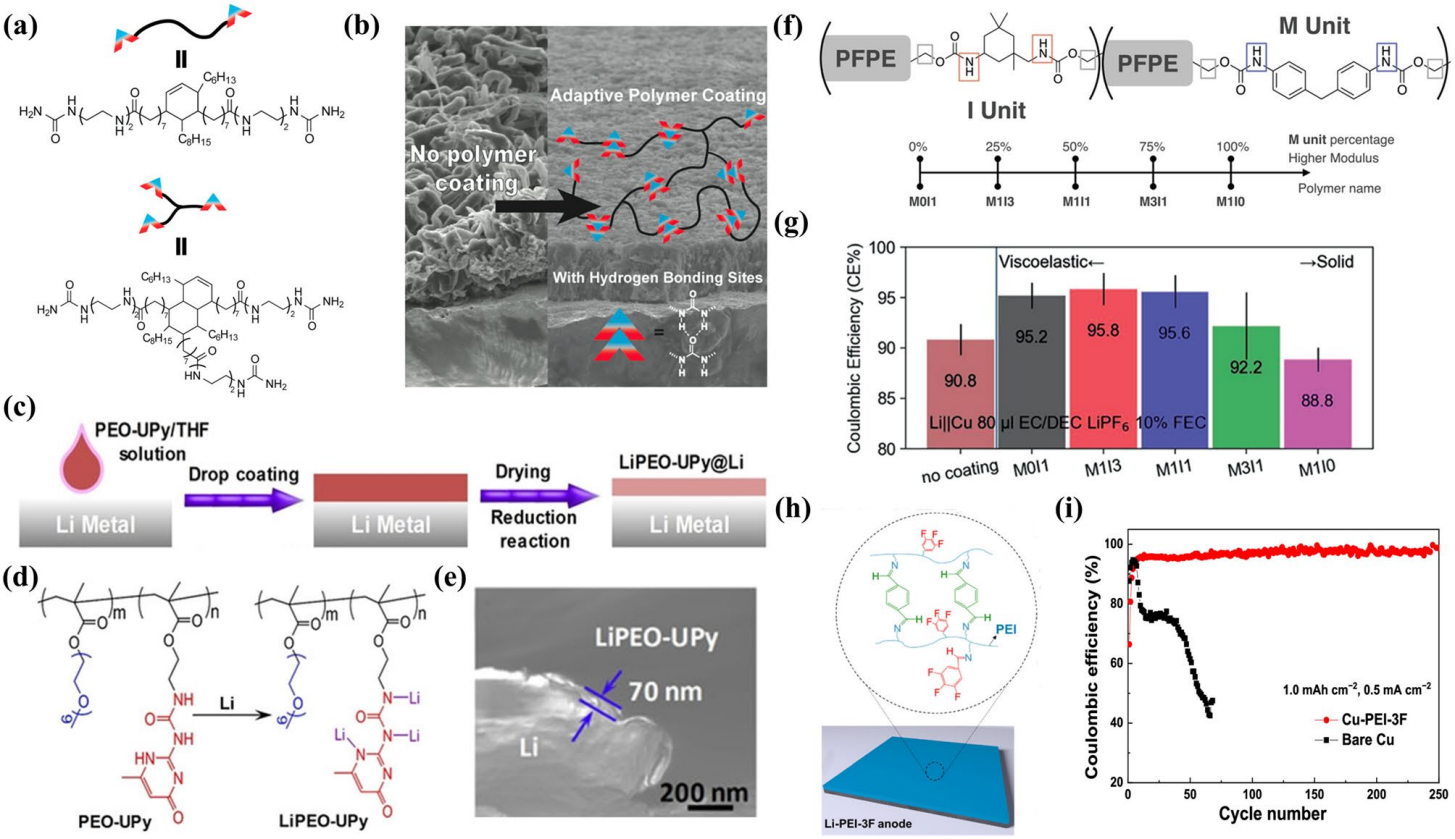
**a** Molecular structure of healing polymer. **b** SEM image comparison of Li deposition morphology (reproduced with permission from Ref. [165]. Copyright 2016, American Chemical Society). **c** Schematic diagram of PEO–UPy coating on Li metal surface. **d** Schematic diagram of the formation of LiPEO-UPy. **e** SEM image of the LiPEO–UPy@Li anode (reproduced with permission from Ref. [166]. Copyright 2020, WILEY–VCH). **f** Structure of polymers with the different strength of hydrogen bond units. **g** CE of electrodeposition (reproduced with permission from Ref. [167]. Copyright 2021, WILEY–VCH GmbH). **h** Schematic diagrams of the preparation of the PEI-3F interlayer on a Li metal anode. **i** Coulombic efficiency *vs* cycle number (reproduced with permission from Ref. [168]. Copyright 2021, American Chemical Society)

Zheng et al. [165] applied highly viscoelastic hydrogen bond polymers to Li anodes (Fig. 10a). The slow flow of the polymer on the electrode surface avoids the “hot spot” where the Li^+^ flux increases rapidly. This artificial polymer layer prevents the formation of cracks or pinholes in the SEI layer. The SEM image shows that uniform Li deposition is achieved at a high current density (Fig. 10b). Wang et al. [166] successfully prepared a novel supramolecular copolymer protective layer containing UPy groups and side chain segments of polyethylene oxide (PEO) by a simple drop coating method (Fig. 10c). After the reduction reaction with Li, the in situ formed compact layer (LiPEO-UPy) can effectively prevent the uncontrollable side reactions and adapt to the huge interfacial volume fluctuations. In addition, the electrostatic interaction between the polar segment of PEO and Li^+^ in the LiPEO-UPy layer can slow down the rapid Li^+^ flux to the Li metal surface, thus enabling uniform lithium deposition (Fig. 10d). The results show that the self-stabilized and compact artificial SEI (~ 70 nm) can eliminate the formation of Li dendrites (Fig. 10e). The above-mentioned polymer is based on weak dynamic interaction, and has high viscoelasticity and weak mechanical toughness, but cannot resist large deformation. Huang et al. [167] designed a healing polymer with different mechanical properties by taking perfluoropolyether (PFPE) with low *T*_g_ as the main chain and regulating the polymerization monomers (isophorone (I) and dimethylenediphenyl units (M)) to change the strength of hydrogen bond units (Fig. 10f). The difference in molecular structure leads to a similar change in mechanical properties. The polymers with high proportion of strong hydrogen bonding units have a more orderly structure, which is characterized by viscous solids, while the other weak hydrogen bonding polymers are characterized by viscoelastic liquids. When these polymers are used as artificial SEIs on Li anodes, the fluidity/softer polymer can achieve high electrodeposition Coulombic efficiency (CE), while the rigidity polymer shows the opposite (Fig. 10g). In addition to non-covalent bond induced self-healing responses, dynamic non-covalent bond also shows certain potential in the design of artificial interface layers. By introducing an intermediate layer of poly (ethylene imine) (PEI) with self-healing and Li adjustment capabilities, Cui et al. [168] can achieve uniform Li deposition and self-healing SEI layer (Fig. 10h). The intermediate layer is cross-linked by C=N bond and contains a trifluorophenyl group. The trifluorophenyl can cooperate with Li^+^, so that the intermediate layer can adjust the Li^+^ distribution at the electrode/electrolyte interface, while the C=N bond enables the artificial layer to a self-healing ability. The resulting Li anode has excellent cycle stability with 250 cycles in an asymmetric Cu | | Li battery and dendrite-free morphology (Fig. 10i).

The self-healing polymer is usually designed with low *T*_g_ temperature, good flexibility and low Young’s modulus to realize rapid molecular identification and recombination, thus effectively responding interface change. However, once the strain limit is exceeded and timely healing is not achieved, the self-healing polymer can no longer provide continuous and stable protection layer. Contrarily, high cross-linking strength will reduce the efficiency of repair. Most polymer interfaces have poor thermodynamic stability in organic liquid electrolytes. The decomposed by -products can cause electronic leakage and then lose the electronic insulation characteristics of the artificial SEI interface. And the lithium-ion diffusion dynamics are also limited due to the low lithium-ion conductivity of polymer. The ideal self-healing artificial interface requires a balance between ionic conductivity, interface compatibility, mechanical strength and chemistry/electrochemical stability. Therefore, new multifunctional self-repairing polymer interface compatible with battery still needs to be further explored.

### Integrated Self-healing

Based on the previous reports, the self-repair function presents obvious locality, that is, only a small part of the device has the repair function. Additionally, the existing synthetic self-healing polymers are only applicable to specific electrolytes or electrode materials. There is still a lack of general-purpose polymers that can manufacture various self-healing electrodes, electrolytes and artificial interface layers. Therefore, realizing the integrated self-healing function of device has received extensive attention.

**Fig. 11:**
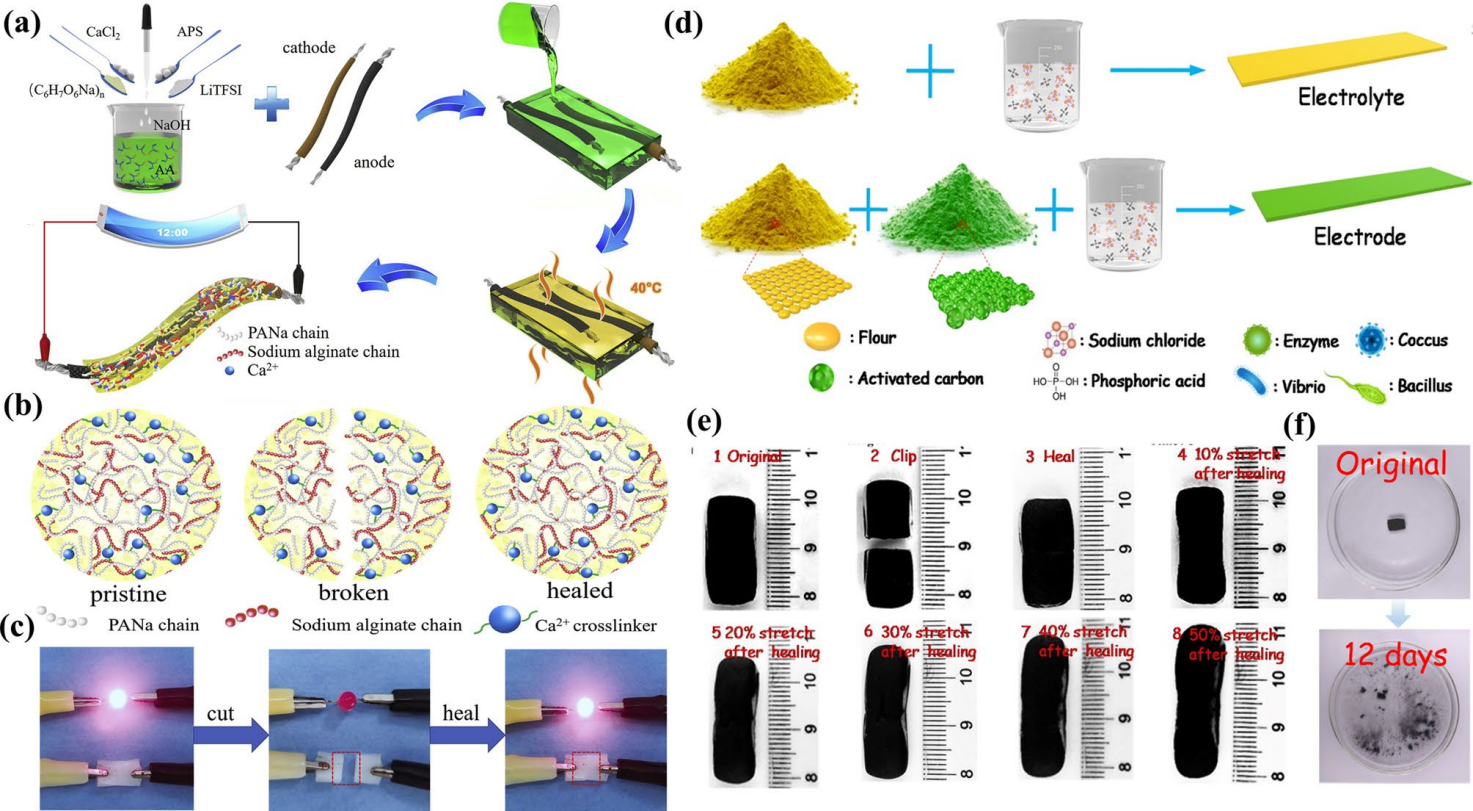
**a** Preparation process of yarn battery based on calcium ion (Ca^2+^) cross-linking. **b** Self-healing mechanism of PANa-Ca-SA hydrogel electrolyte. **c** Cutting/healing experiment (reproduced with permission from Ref. [169]. Copyright 2020, Elsevier). **d** Schematic diagram of the preparation of a one-stop supercapacitor. **e** Tensile test. **f** Biodegradation experiment (reproduced with permission from Ref. [170]. Copyright 2018, Elsevier)

Based on the calcium ion (Ca^2+^) cross-linked double network of sodium polyacrylate and sodium alginate (PANa-Ca-SA) hydrogel electrolyte, Ji et al. [169] prepared an ultra-long life aqueous lithium-ion yarn battery (ALIYB) with microscopically and macroscopically self-healing properties (Fig. 11a). When the broken hydrogel contacts again along the crack, the broken chain will be re-connected through Ca^2+^ cross-linking (Fig. 11b). Meanwhile, the electrode material is in situ polymerized in the PANa-CA-SA hydrogel electrolyte with self-healing ability, which can effectively prevent the initiation and expansion of microscopic cracks on the electrode. The cutting/healing experiment shows that the hydrogel could not light up the red light-emitting diode (LED) after cutting (Fig. 11c). When the hydrogel re-contacted, the red LED would immediately light up again. However, the supercapacitors are not available for stretching due to the limited self-repair effect. Different from the previous reports, Hu’s research team [170] proposed a new concept of biodegradable and one-stop self-healing supercapacitor, using modified flour as the additive material for electrodes and electrolytes (Fig. 11d). The hydrogen bonds between flour and water molecules can ensure the simultaneous repair of electrodes and electrolytes. Importantly, supercapacitor can be stretched essentially from 0 to 50% without fracture, which has never been realized in the past reports (Fig. 11e). Besides, the supercapacitor can be degraded by microorganisms, thus providing a unique solution for the disposal of recycling supercapacitors (Fig. 11f).

The integrated self-healing devices can favorably adapt to flexible application scenarios, avoid the single healing limitations and interface incompatibility between different components. However, the currently integrated self-repair application is only limited to hydrogel systems or water-based batteries with low mechanical strength and energy density, which cannot meet the requirements of practical applications. Therefore, reasonable design and building integrated self-repairing functions are currently the main research tasks to meet a variety of battery or capacitor system requirement.

## Advanced Self-healing Characterization Technology

In addition to emphasizing self-healing mechanism and efficiency, advanced characterization techniques are very important to analyze structure and composition of self-healing materials and understand self-repair mechanism, which is the key to promoting the development of self-repairing chemistry. Herein, we mainly introduce two parts including chemical structure and visualization characterization.

### Chemical Structure Characterization

Fourier transform infrared spectroscopy (FTIR) [171–173] and nuclear magnetic resonance spectroscopy (NMR) [174, 175] are two key technologies for characterizing the chemical structure information of self-healing materials. FTIR is the most commonly used characterization method in the field of self-healing materials, which can detect the functional groups of unknown substances, determine the chemical structure and observe the chemical reaction process according to the different spectrum characteristics. NMR can analyze the materials’ fine structure and determine the local chemistry information of reactants or products, providing a favorable mean for in-depth understanding of the self-repair mechanism.

**Fig. 12:**
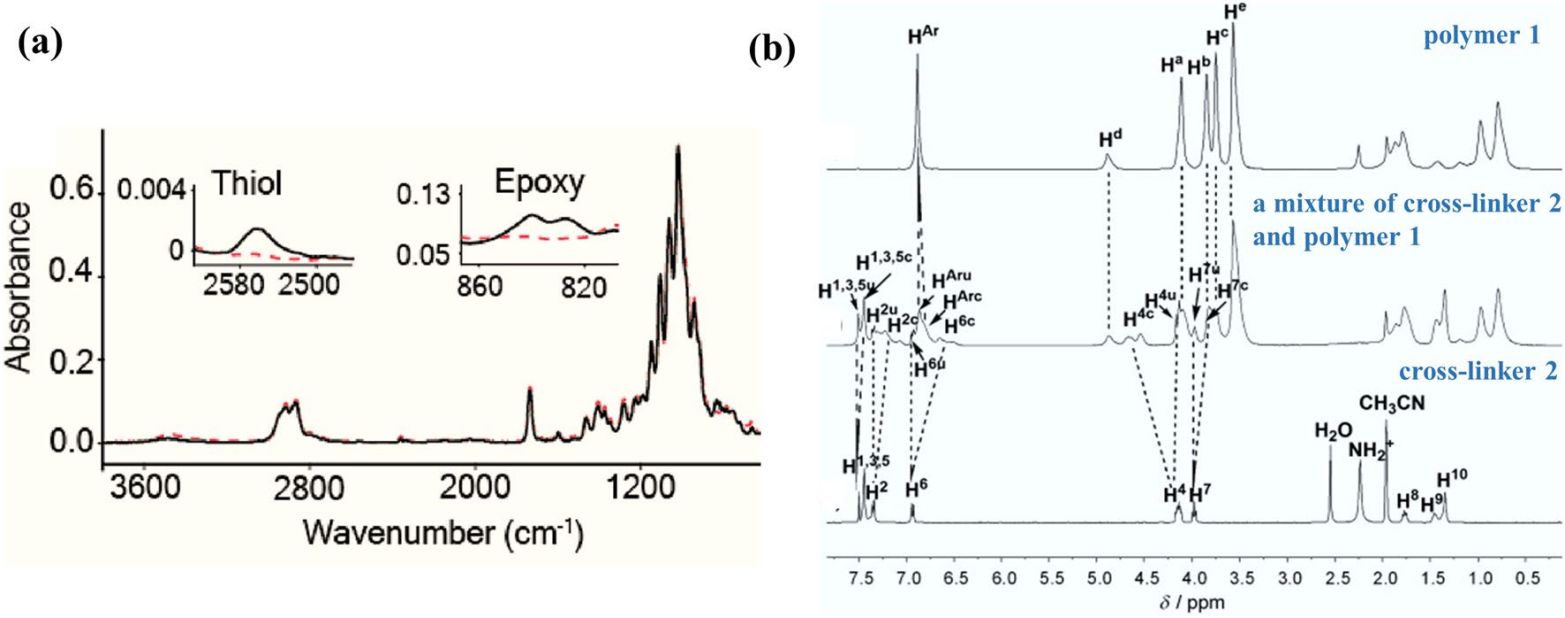
**a** FTIR/ATR spectra of cross-linking process (reproduced with permission from Ref. [176]. Copyright 2011, American Chemical Society). **b** Partial ^1^H NMR spectra (reproduced with permission from Ref. [177]. Copyright 2012, WILEY–VCH)

In Canadell’s work, the cross-linking process of epoxy resin and mercaptan in alkali catalyzed addition reaction was monitored by FTIR. The complete disappearance of the tensile vibration peaks of ethylene oxide (860–815 cm^−1^) and the S–H tensile vibration peaks of mercaptan groups (2536 cm^−1^) indicates that the conversion rate of the cross-linking reaction is close to 100% and the formation of self-healing disthionyl groups (Fig. 12a) [176]. Zhang et al. [177] used NMR to understand the complexation behavior of polymers. As shown in Fig. 12b, polymer 1 can cross-link agents 2 and 3 in solution. The aromatic protons H^1^, H^2^, H^3^, H^5^, H^6^ and the phenoxymethylene protons H^7^ of 2 showed an upfield chemical shift, while the downfield chemical shift of benzyl proton H^4^ after complexation indicated the formation of host–guest interactions. The chemical structure analysis reliability is closely related to the characterization accuracy. Developing high-resolution test methods is very necessary for understanding self-healing evolution.

### Visual Self-healing Characterization

**Fig. 13:**
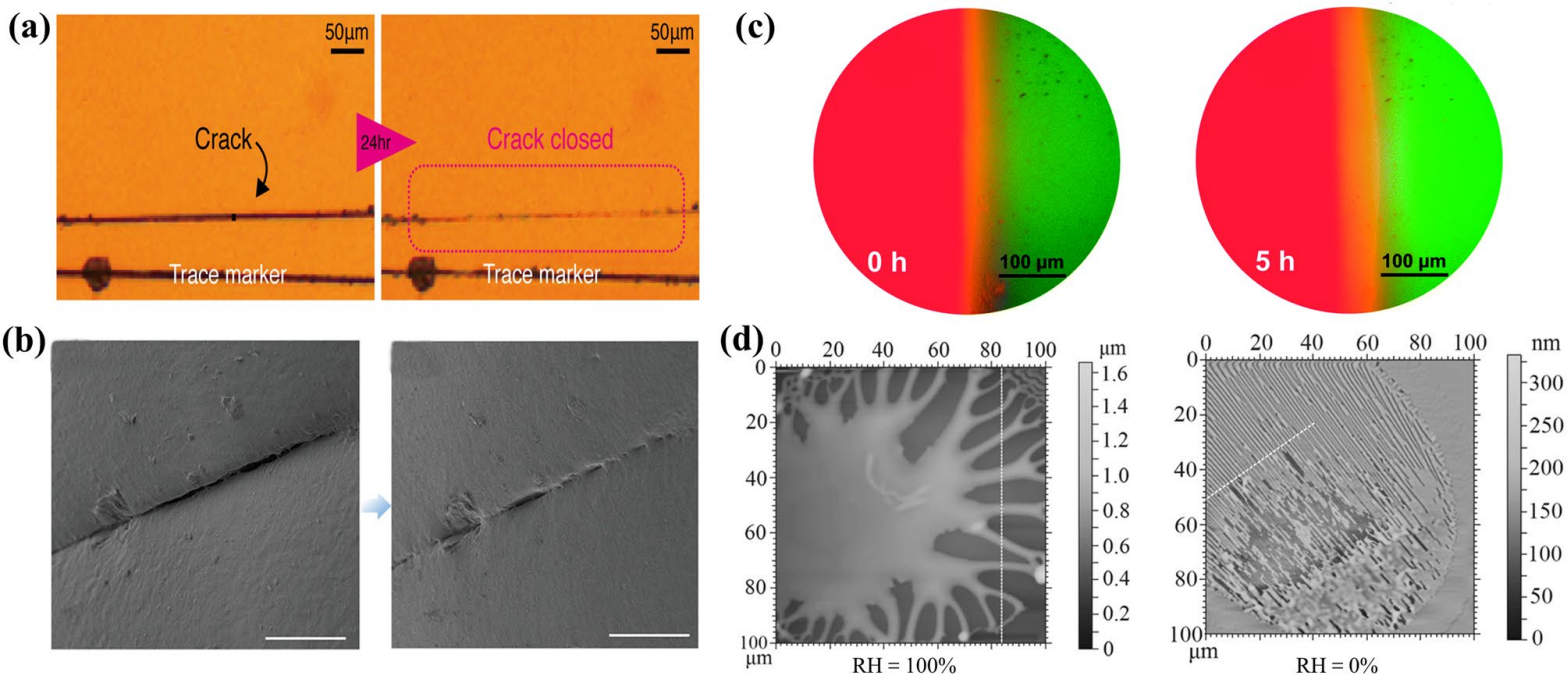
**a** Optical microscope images of Fe-PDBP@pH10 film before and after 24 h (reproduced with permission from Ref. [131]. Copyright 2019, American Chemical Society). **b** Self-healing SEM images of the CPE surface (reproduced with permission from Ref. [164]. Copyright 2019, The Authors. Published by WILEY–VCH). **c** Fluorescence microscopy images illustrating the self-healing process of SHSPE3 (reproduced with permission from Ref. [144]. Copyright 2021, Royal Society of Chemistry). **d** AFM images in contact mechanics tests (reproduced with permission from Ref. [178]. Copyright 2014, WILEY–VCH)

Visual characterization of the healing process can deepen the understanding of the self-healing mechanism, but the current monitoring methods are often limited. Microscopes are commonly used to monitor self-healing behavior according to the size and change of morphology, such as optical microscopy (OM) [131, 138], SEM [135, 164] and fluorescence microscopy [144]. Researchers use these techniques to investigate the healing characteristics including the morphology evolution of polymer film (Fig. 13a) [131], the crack size reduction of electrolyte film (Fig. 13b) [164] and the polymer segments mutual diffusion in the fluorescence image (Fig. 13c) [144], but these macroscopic characterization techniques are limited to polymer surface structures, and the internal self-repair behavior is often not observed. Besides, atomic force microscopy (AFM) has been used to analyze self-healing behavior. Faghihnejad et al. [178] used AFM to characterize the relative humidity (RH) of self-healing polymers with multiple hydrogen bonds (Fig. 13d). When RH = 0%, the surface pattern is in the form of linear and almost parallel bands or stripes, while at RH = 100%, a larger branched radial finger is formed, indicating that the transition from elastic failure to viscous failure occurs in the polyhydrogen-bonded polymer when RH = 0% increases to RH = 100%. Visual characterization methods with high temporal and spatial resolution are still to be applied in the field of self-healing chemistry, especially some in situ synchrotron radiation-based characterization tools.

## Conclusions and Perspective

With the increasing global environmental and energy crisis, the development of energy storage devices is in full swing. Batteries or supercapacitors for various application scenarios have received extensive attention from both industry and academia, especially for flexible electronics. Mechanical flexibility can enable electronic devices to achieve wearable functions. Meanwhile, electrochemical stability and safety of flexible batteries or supercapacitors under mechanical deformation are still a formidable challenge. Self-healing chemistry provides a proven means to realize excellent electrochemical performance of flexible electronic devices. Self-healing means that the mechanical damage can be repaired in time, and the electrochemical performance can be restored as before. Therefore, realizing self-healing energy storage device is a very promising strategy to promote the further development and application of flexible electronics.

### Conclusions

**Fig. 14:**
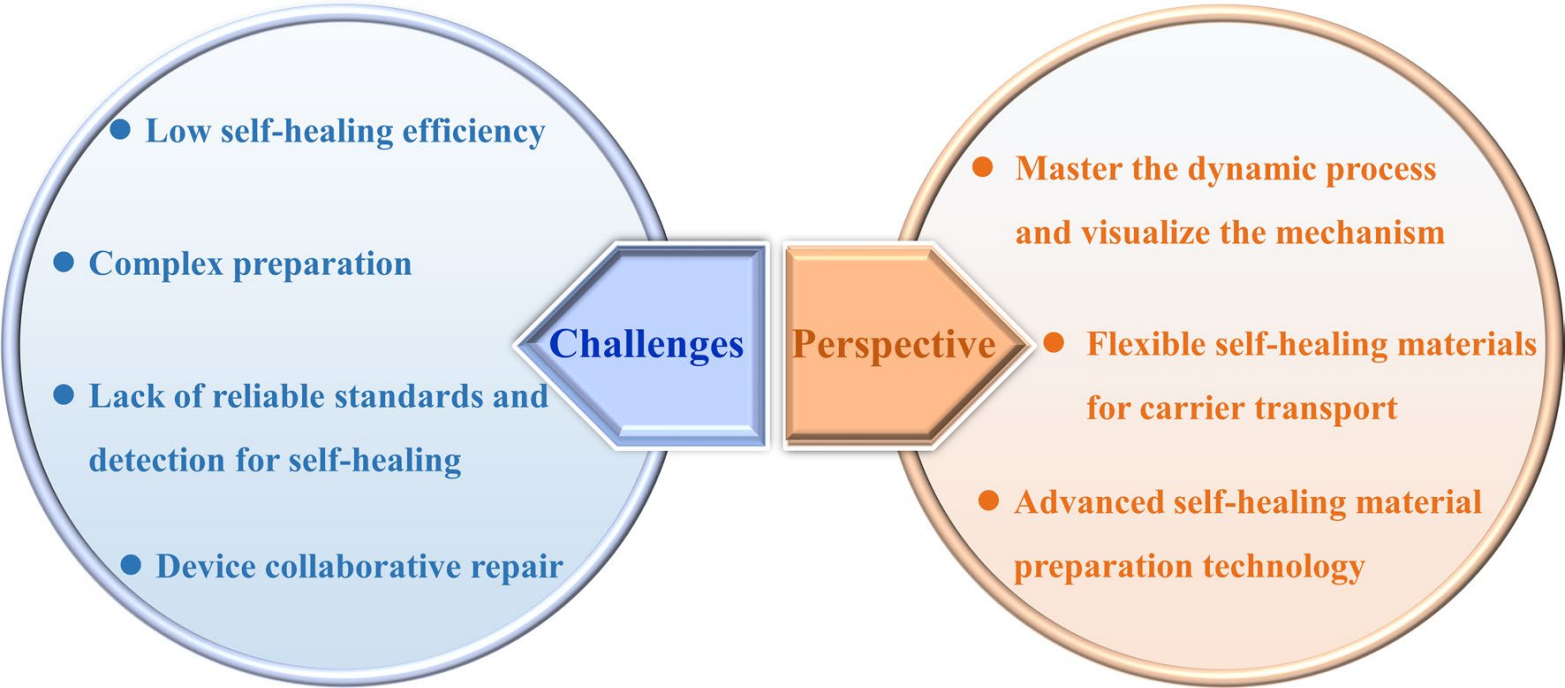
Challenges and perspective for self-healing energy storage

At present, although a series of researches on self-healing flexible energy storage devices have been carried out, some key challenges remain (Fig. 14): (1) The current self-healing efficiency based on various bonds is still limited and not ideal. The low self-healing ability directly leads to the poor electrochemical performance of flexible devices. Moreover, some self-healing effects require certain external stimuli and drives, for example, dynamic covalent bond-driven self-healing. It is necessary to further develop novel self-healing materials to ensure high repair efficiency and stable electrochemical performance recovery of energy storage devices. (2) The current self-healing is mostly unilateral repair of electrodes or electrolytes. The research of comprehensive self-healing mainly focuses on water system. Self-healing polymers developed at present are only applicable to specific electrolytes or electrode materials. Synergistic polymers that can be used to make a variety of self-healing electrolytes and electrodes are still lacking. (3) The self-healing material preparation and their integration with batteries or capacitors are still complex and cumbersome, which is extremely unfavorable for large-scale commercial production of self-healing energy storage. Therefore, it is necessary to make great progress in simplifying the preparation process and obtaining excellent self-healing materials. (4) There is a lack of reliable standards and guidelines for the evaluation and detection of self-healing functions. For example, self-healing efficiency refers to the speed at which the polymer fracture surface repairs the damage through its own physical or chemical interactions, the determinant of which is the factor of chain diffusion and chemical recombination of polymer molecules. Currently, the healing efficiency can be defined in terms of fracture toughness, tensile strength or Young’s modulus of polymers. For the evaluation of healing efficiency in electrochemical energy storage, most methods are damage/healing experiments. However, more complex electrochemical reactions are usually involved in energy storage devices, so it is more urgent to understand the scientific problems of healing in the process of charging/discharging on a microscopic scale.

### Perspective

Based on the current achievements, the following research directions are proposed for the application of self-healing in the next-generation flexible energy storage devices (Fig. 14). (1) Most reported self-healing materials are far from practical application. To meet the needs of the highly flexible/wearable devices, the exploration and preparation of polymers with strong mechanical strength and high repair efficiency play a key role in promoting self-repairing flexible energy storage devices. One of the ways to solve the contradiction between mechanical strength and self-repair is to use the synergy of double dynamic action (strong and weak mixing) and micro-phase separation (hard phase locking). In addition, the fusion of different advanced material preparation technologies (response driven (thermal initiation, ultraviolet light initiation, magnetic initiation, pH value initiation, etc.), atomic layer deposition and electrospinning, etc.) is a new way to simplify the preparation process to obtain excellent materials. (2) Most self-healing polymers are insulated, and their applications are mostly independent. The ideal self-repair requires not only the mechanical recovery, but also the autonomous recovery of electrochemical activity. New flexible self-healing materials capable of carrier transport are needed to replace traditional electrolytes or fluid collecting materials. Currently available functional materials such as liquid metal and conductive self-healing polymers, combined with 3D printing and winding preparation processes, are conducive to the development of self-healing. (3) Master the dynamic process of healing and understand the working mechanism. The development route of flexible energy storage device needs to consider the stability of electrode and electrolyte. Interface layering and mechanical damage between components of devices remains a challenge due to the differences in mechanical properties between components. Therefore, combined with advanced in situ characterization techniques (synchrotron radiation, in situ nuclear magnetic resonance) and electrochemical analysis, the observation and exploration of the interfacial changes of the chemical composition and microstructure will contribute to a better understanding of the failure mechanism of the device. Simplify the preparation process of self-healing materials, optimize and develop a new universal self-healing system, and realize the self-diagnosis process of material damage, so as to realize the real intelligent bionic self-healing flexible energy storage.
